# Mapping and conservation analysis of upper montane evergreen broad‐leaved forest using machine learning and geodata

**DOI:** 10.1002/eap.70333

**Published:** 2026-10-22

**Authors:** Wenqi Zhang, Yanxia Wang, Yuhang Zhou, Yuanbei Liu, Weihong Zeng, Ruliang Zhou

**Affiliations:** ^1^ College of Soil and Water Conservation Southwest Forestry University Kunming Yunnan China

**Keywords:** conservation management, habitability probability modeling, machine learning, monsoon circulation, upper montane evergreen broad‐leaved forest (UMEBLF)

## Abstract

The upper montane evergreen broad‐leaved forest (UMEBLF) provides unique ecological functions but exhibits a highly fragmented spatial distribution. Mapping the suitable habitat of UMEBLF and assessing its conservation status are therefore crucial for regional protection and management of this vegetation. In this study, Yunnan Province, China, was used as a case study and employed 11 topographic and climatic variables, including mountain relative height, to identify an optimal Random Forest model for simulating and mapping the suitable distribution of UMEBLF under current and future climate scenarios. In addition, the montane cloud–fog belt within the monsoon core region was incorporated to evaluate the reliability of the mapping results under current climatic conditions. By comparing the spatial distribution of nature reserves across the province, the current protection status of UMEBLF and its potential future changes were clarified. Results indicate that under current climatic conditions, areas with high habitat suitability for UMEBLF cover approximately 4.2 × 10^4^ km^2^, primarily distributed across the mid‐montane zones of western and central Yunnan. The lower boundary of suitable habitat closely coincides with the elevation of the cloud–fog belt and is primarily located on middle to upper slopes, at approximately two‐thirds of the mountain's relative height above the base surface. Under future warming scenarios, the spatial pattern of suitable habitat exhibits relatively stable core areas and highly dynamic marginal zones. Under the SSP5‐8.5 scenario, the area of highly suitable habitat is projected to decline to approximately 3.0 × 10^4^ km^2^ by the 2090s. Currently, 32 nature reserves contain areas with high habitat suitability, with 75% concentrated on the southwestern side of the Cangshan–Ailao Mountain region. In the future, the proportion of highly suitable habitat areas within protected zones is expected to decrease by 15.9%–18.7%, with reductions occurring mainly within experimental zones of nature reserves. This study demonstrates an integrated framework combining geospatial big data and machine learning to map and analyze UMEBLF distribution, providing a scientific basis for mountain forest conservation and management under climate change.

## INTRODUCTION

In the context of global climate warming, vegetation mapping and conservation analysis are of substantial practical importance for biodiversity protection. The upper montane evergreen broad‐leaved forest (UMEBLF) in Yunnan represents an ecologically important and unique forest type within subtropical montane ecosystems (Liu et al., 2021; Zhu et al., 2016). In recent years, climate warming, a reduction in foggy days, and increasing drought intensity, combined with the expansion of agroforestry under China's rural revitalization strategy, have subjected species within this forest type to increasing environmental stress. These combined pressures have markedly increased the risk of habitat degradation, particularly in marginal areas (Bruijnzeel, Mulligan, & Scatena, 2011; Foster, 2001; Liu et al., 2004, 2021; Yang et al., 2024). However, most existing studies are site‐based and focus primarily on community characteristics and species composition (Meng et al., 2013; Zhu et al., 2016), while comparatively limited attention has been given to the spatial distribution of suitable habitat and the protection status of UMEBLF (Feeley & Silman, 2010; Zhu et al., 2016). This knowledge gap poses challenges for conservation planning under a warming climate. Therefore, assessing the suitable distribution and conservation status of UMEBLF is of great significance for protecting this unique forest type and maintaining ecosystem security in low‐latitude plateau regions (Brooks et al., 2002; Feeley & Silman, 2010; Ponce‐Reyes et al., 2013; Watson et al., 2014; Zhu et al., 2016).

UMEBLFs are also referred to as cloud forests or mossy forests in Asia (Shi & Zhu, 2009) and represent a characteristic vegetation subtype within the vertical zonation of subtropical montane regions. Their global distribution is relatively limited, with core areas located in the Ailao and Gaoligong Mountains of Yunnan, China, as well as along the southern slopes of the Himalayas in Nepal, Bhutan, and northeastern India. Classical studies suggest that warm and humid montane habitats formed under the combined influence of orographic rainfall and local environmental conditions have promoted the development of this forest type across the subtropical mid‐montane regions of Yunnan, generally below elevations of 2900 m (Jin, 1979; Wu, 1987). UMEBLFs typically occur within the rain–fog belt and differ markedly in floristic composition and physiognomy from the semi‐humid evergreen broad‐leaved forests and monsoon evergreen broad‐leaved forests found in the low mountain and hilly zones of the same plateaus. Zhu et al. (2016) proposed standardizing the term “mid‐montane wet evergreen broad‐leaved forest” as “UMEBLF,” and this terminology is adopted in the present study.

UMEBLF integrates the climate–soil environment and vegetation ecosystem characteristics of the mid‐montane zones of the Yunnan Plateau and holds substantial value for vegetation ecology research and environmental conservation. In Yunnan, extensive areas of UMEBLF are currently preserved within national nature reserves such as the Gaoligong Mountain, Ailao Mountain, and Wuliang Mountain reserves, as well as provincial nature reserves including Yongde Daxueshan, Tongbiguan, and Daweishan (Yunnan Forestry and Grassland Administration, 2020). Nevertheless, the expansion of rubber and tea plantations outside protected areas, together with the barrier effects of highways and other infrastructure, has intensified the insular and belt‐like fragmentation of UMEBLF distribution (Laurance et al., 2014; Xu et al., 2014). Owing to its high sensitivity to both climate change and human disturbance, UMEBLF has become one of the vulnerable forest types that urgently require assessment and targeted conservation under changing climatic conditions.

Traditional investigations of UMEBLF have relied mainly on field surveys supplemented by remote‐sensing interpretation, using transect or plot‐based sampling to collect vegetation and species data over large areas. This approach is both labor‐intensive and expensive and often requires several years to complete (Tang et al., 2025; Xu et al., 2025). To overcome these constraints, spatiotemporal simulation methods have been adopted in several studies. By integrating site‐based observations, remote‐sensing inversion, and geographic information system‐based data assimilation, field sampling requirements are reduced, and the development of probabilistic vegetation distribution models to support mapping and conservation planning is facilitated. In recent years, the Biomod2 platform, which integrates multiple modeling approaches, has been widely applied in species distribution modeling (Merow et al., 2013; Phillips et al., 2006; Thuiller et al., 2019), gradually replacing the previously dominant MaxEnt model (Merow et al., 2013; Phillips et al., 2006; Thuiller et al., 2019). This transition reflects the growing recognition that machine learning approaches are better suited to simulating and predicting the complex nonlinear interactions among multiple environmental factors that shape UMEBLF distribution patterns. Moreover, the sparse and fragmented distribution of UMEBLF limits sample availability, rendering deep learning models that require large training datasets impractical (Chen & Guestrin, 2016; Thuiller et al., 2009). Consequently, the selection of an appropriate machine learning model to predict the distribution of suitable UMEBLF habitats represents a feasible pathway for mapping and forecasting UMEBLF distribution (Breiman, 2001; Chen & Guestrin, 2016).

Geospatial big data provide essential support for applying machine learning models to simulate potential species distributions. Localized variability in temperature and humidity gives rise to banded and fragmented cloud–fog belts, within and along whose upper boundaries temperature inversions frequently occur and relative humidity remains persistently high (Muñoz‐Villers et al., 2012). Theoretically, the boundary of suitable UMEBLF habitat should therefore exhibit a strong spatial correspondence with the cloud–fog belt (Muñoz‐Villers et al., 2012). However, conventional topographic variables, such as elevation and slope aspect, primarily describe absolute position and are insufficient for capturing the “relative position” that influences vegetation distribution. Recent studies have not only delineated cloud–fog belts at regional scales using meteorological and remote sensing data but have also developed new geoscientific variables that describe relative position, including relative height above or below watershed reference surfaces, mountain base elevation, and relative slope height. These novel geospatial datasets more effectively delineate boundaries influenced by warm and humid airflows (Wang et al., 2024), particularly in situations where climate anomalies induced by warm–humid airflows are more pronounced than terrain‐driven redistribution effects. Incorporating such variables into machine learning modeling and validation processes can enhance both predictive accuracy and explanatory power of UMEBLF distribution simulations (de Reu et al., 2013; Gallant & Dowling, 2003; Jasiewicz & Stepinski, 2013; Karger et al., 2017; Nobre et al., 2016).

In this study, bioclimatic data and newly developed geoscientific geospatial datasets were used to identify the optimal model from six mainstream machine learning algorithms for mapping the suitable distribution of UMEBLF and assessing its protection status under current climatic conditions and future climate scenarios. First, partial correlation analysis and Gini importance analysis were employed to screen and interpret the independent effects of environmental factors and their contributions to the model. Meanwhile, principal components analysis (PCA) was used to evaluate the representativeness of the presence samples with respect to key environmental variables. Second, the predictive performance of different models was compared using metrics such as the area under the receiver operating characteristic curve (AUC), the true skill statistic (TSS), and the F1 score, and the robustness of the optimal model under the selected presence samples was further tested through repeated subsample modeling. Subsequently, the suitable habitats of UMEBLF were simulated and mapped under current climatic conditions and under three future carbon‐emission scenarios across two future periods, and the ecological plausibility of the boundary of the Optimally Suitable areas under current climatic conditions was evaluated using the boundary of the montane cloud–fog belt. Finally, by overlaying the spatial boundaries of nature reserves, the protection status of UMEBLF and its potential changes under current and future climatic conditions were analyzed.

## MATERIALS AND METHODS

### Study area

Yunnan Province is located between 21.14°–29.23° N and 97.54°–106.20° E (Figure 1). Mountainous terrain accounts for approximately 63.5% of the total area of the province, and this is characterized by high mountains, steep slopes, and deep valleys (Ye et al., 2025). Under the combined influence of warm and humid airflows associated with the southwest monsoon, high mountain ranges, and deeply incised valleys, abrupt changes in temperature and humidity are common across the region (Yang et al., 2025; Ye et al., 2025; Zhu et al., 2019).

**FIGURE 1:**
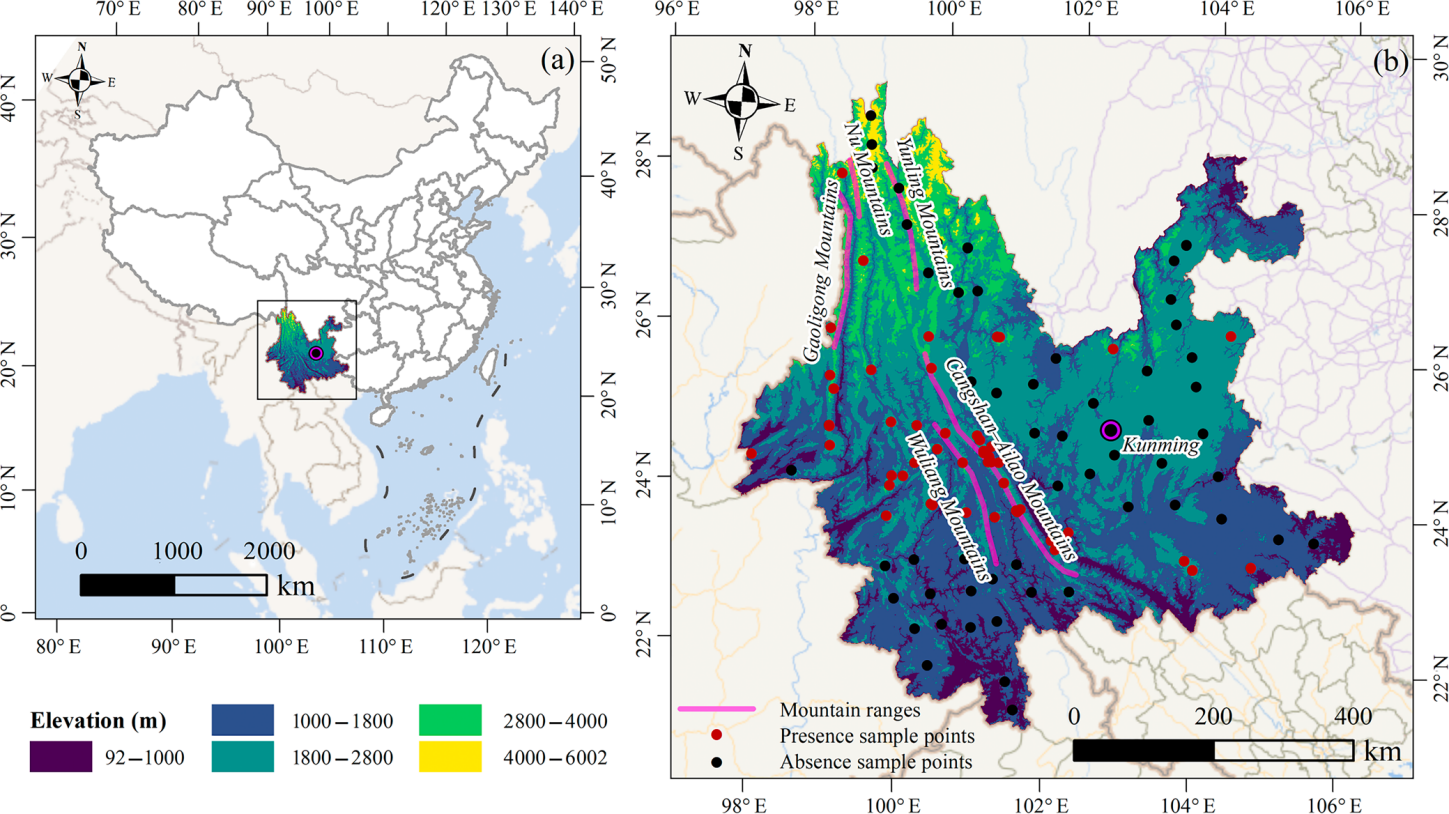
(a) Geographic location of Yunnan Province in China; and (b) distribution of presence and absence sample points of upper montane evergreen broad‐leaved forest (UMEBLF) in Yunnan. This map is based on the standard map provided by the Map Review Center of the Ministry of Natural Resources of China (Map Review Number: GS(2024)0650), with no modifications to the base map. The same applies to all subsequent maps.

In particular, high mountain bodies often exhibit two banded zones of abrupt temperature and humidity transition. One such zone is located in the transitional region from the valley bottom to the lower mountain slope, and the other between the middle–upper slopes and the mountain crest (Wang et al., 2024). In Yunnan, UMEBLF is mainly distributed in the mid‐montane zones of several major mountain ranges on the southwestern side of the central Yunnan Plateau, including the Ailao and Wuliang Mountains, as well as in the Gaoligong Mountains of western Yunnan (Tang & Ohsawa, 2009; Yu & Qian, 2017; Zhu et al., 2019).

Frequent cloud and fog events characterize the UMEBLF distribution area. Winters are generally mild and summers are cool, with mean annual temperatures of 10–15°C, annual precipitation exceeding 1200 mm, and relative humidity consistently above 80% throughout the year (Tang & Ohsawa, 2009; Yang et al., 2025; Zhu et al., 2019). The forest exhibits high floristic diversity, dominated by evergreen broad‐leaved tree species belonging mainly to the families Fagaceae, Theaceae, and Lauraceae (Tang & Ohsawa, 2009; Yu & Qian, 2017; Zhu et al., 2019).

### Data sources and processing

#### Known geographic distribution data

Geographic distribution records of UMEBLF were compiled from herbarium records, published literature, and field surveys (Eckert et al., 2024; Li et al., 2025; Ni, 2023; Shi et al., 2025; Zhang et al., 2016; Song et al., 2021; Zhou et al., 2023). To minimize the influence of spatial autocorrelation on model construction, records were spatially filtered and thinned such that only one presence point was retained within each 1 km × 1 km grid cell. When multiple records occurred within a single grid cell, the point closest to the grid center was retained. A total of 54 UMEBLF presence points were obtained. Following the disk strategy proposed by Barbet‐Massin et al. (2012), 54 absence points were randomly generated at locations at least 10 km from all presence points to represent non‐suitable environments. This yielded 108 sample points in total (Figure 1b), of which 70% were used for model training and 30% for testing.

#### Climate data

Both current and future climate variables were obtained from WorldClim version 2.1 (Fick & Hijmans, 2017). After excluding variables with correlation coefficients greater than 0.8 (Dormann et al., 2013), seven bioclimatic variables were retained: BIO1, BIO2, BIO3, BIO4, BIO12, BIO14, and BIO15. These variables represent thermal, moisture, and radiation conditions relevant to vegetation growth. Current climate data represent the average conditions during 1970–2000. Future climate projections were derived from the Beijing Climate Center Climate System Model (BCC‐CSM2‐MR) (Wu et al., 2019), which shows stable performance and strong capability in simulating Asian summer monsoon precipitation. Three emission scenarios (SSP1‐2.6, SSP3‐7.0, and SSP5‐8.5) were selected for two future periods, namely 2041–2060 (2050s) and 2081–2100 (2090s). Detailed information on climate data is provided in Table 1.

**TABLE 1:** Data sources and introduction.

Factor type	Code	Description	Data source	Unit
Meteorological Factor	BIO1	Annual mean temperature	Mean for 1970–2000, WorldClim	°C
	BIO2	Mean diurnal range	Mean of monthly (max temp − min temp), WorldClim	°C
	BIO3	Isothermality	(BIO2/BIO7) × 100, WorldClim	…
	BIO4	Temperature seasonality	Standard deviation × 100, WorldClim	°C
	BIO12	Annual precipitation	WorldClim	mm
	BIO14	Precipitation of the driest month	WorldClim	mm
	BIO15	Precipitation seasonality	Coefficient of variation, WorldClim	…
Geological Factor	DEM	Digital elevation model	WorldClim v2.1 (SRTM‐derived)	m
	DMTZ	Geomorphological features	Cheng and Zhou (2014), TPDC	…
	TUHCD	Topographic uplift height and cutting depth	Wang et al. (2024)	m
	MRAI	Mountain relative altitude index	Standardized TUHCD	%

Abbreviations: DEM, digital elevation model; DMTZ, digital morphological terrain type; MRAI, mountain relative altitude index; SRTM, Shuttle Radar Topography Mission; TUHCD, topographic uplift height and cutting depth.

#### Geographical environmental data

Geographical variables included the digital elevation model (DEM), digital morphological terrain type (DMTZ), topographic uplift height and cutting depth (TUHCD), and the mountain relative altitude index (MRAI). DEM data were obtained from the WorldClim version 2.1 elevation dataset, which is based on Shuttle Radar Topography Mission (SRTM) elevation data (Fick & Hijmans, 2017). DMTZ data were obtained from the Geomorphological of China 1:4,000,000 dataset provided by the National Tibetan Plateau/Third Pole Environment Data Center (Cheng & Zhou, 2014). Using DEM as the base dataset, a terrain trend surface (TTSA) was constructed, and TUHCD was calculated by subtracting TTSA from DEM (Wang et al., 2023). MRAI represents a standardized form of TUHCD, with values ranging from −100 to 100, where values close to 100 indicate mountain ridges and values near −100 represent valley bottoms. Detailed descriptions of these variables are provided in Table 1.

#### Cloud–fog belt data

The transient humid altitudinal cloud (THAC) phenomenon is widely distributed across mountainous surfaces. Using moderate resolution imaging spectroradiometer infrared bands in combination with SRTM DEM data, a temperature lapse rate (TLR) field was calculated with a sliding window of 3 × 3 cells. Pixels with lapse rate values greater than −0.65°C per 100 m were classified as THAC zones (Wang et al., 2024). THAC zones covering Yunnan Province were obtained from previous research (Wang, 2023) and are publicly available at https://doi.org/10.4121/ba236ea7‐650c‐4bde‐859b‐f6b08b5c22ac.v1.

#### Nature reserve boundary data

Nature reserve boundary data were obtained from the National Nature Reserve Boundary Dataset provided by the Resource and Environmental Science Data Platform (RESDC; https://www.resdc.cn/data.aspx?DATAID=272).

### Research framework

The objective of this study was to develop a machine learning‐based framework for mapping the suitable distribution of UMEBLF and assessing its conservation status. The framework consists of three main components: optimal model construction, suitable habitat mapping, and conservation analysis. First, UMEBLF occurrence data were compiled, and a multisource geospatial dataset including topographic and bioclimatic variables was established. Correlation analysis, partial correlation analysis, and PCA were then applied to evaluate the effectiveness of environmental variables and the representativeness of the presence samples with respect to the major environmental gradients. Subsequently, six mainstream machine learning models were constructed, and the Random Forest (RF) model was selected as the optimal model. On this basis, repeated subsample modeling was further conducted to test the robustness of model predictions to the limited number of presence samples. The selected RF model was used to simulate and predict suitable habitat distribution under current climatic conditions and three future climate scenarios across two future periods, followed by suitable habitat mapping. The montane cloud–fog belt was used to verify the ecological plausibility of the simulated suitable distribution under current climatic conditions. Finally, overlay analysis with nature reserve boundaries was conducted to evaluate the current conservation status of UMEBLF and to assess potential changes under future climate scenarios. The overall technical workflow is illustrated in Figure 2.

**FIGURE 2:**
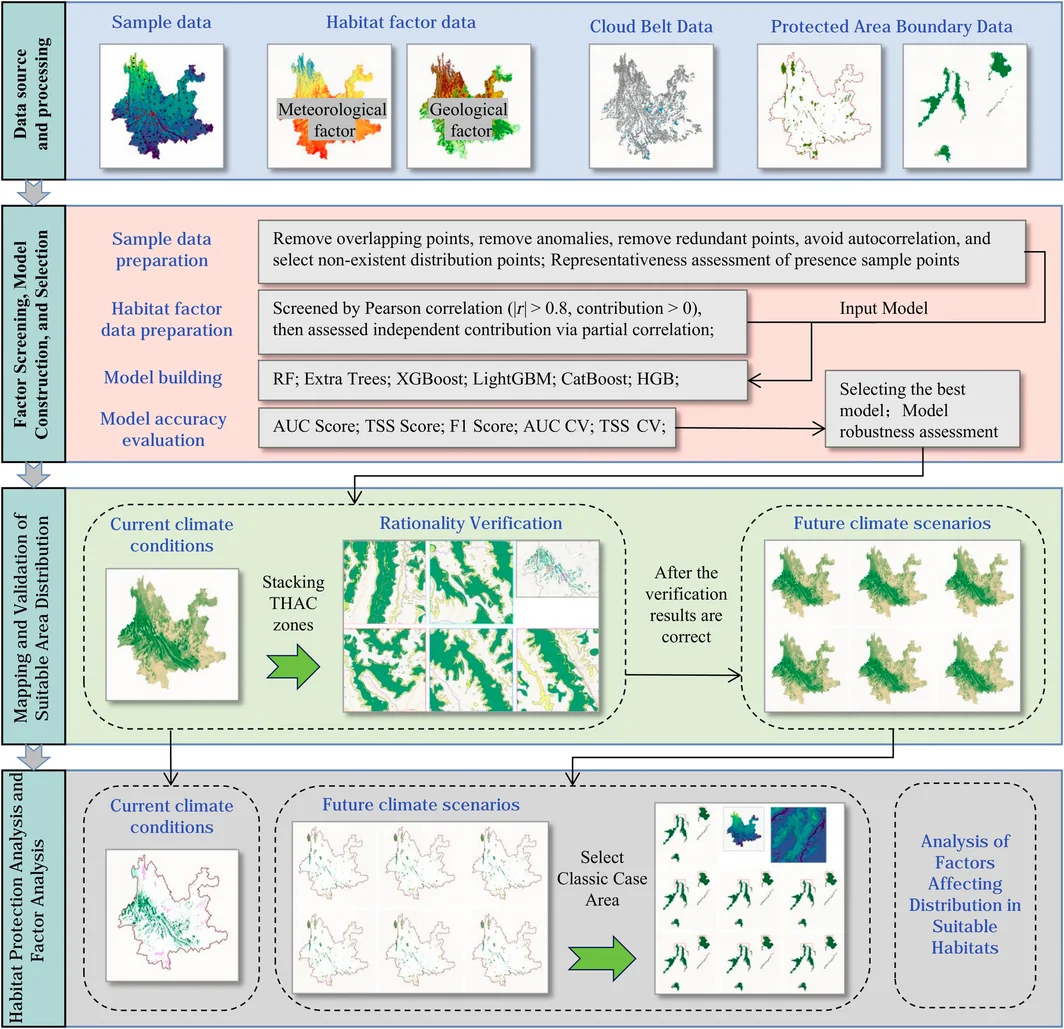
Technical framework of the study. AUC, area under the receiver operating characteristic curve; CatBoost, CatBoost classifier; ExtraTrees, Extremely Randomized Trees; HGB, Histogram‐Based Gradient Boosting; LightGBM, Light Gradient Boosting Machine; RF, Random Forest; TSS, true skill statistic; XGBoost, Extreme Gradient Boosting.

### Optimal model construction

#### Variable analysis

Partial correlation analysis was used to quantify the independent contribution of each variable after controlling for the effects of others (Kim, 2019). As shown in Figure 3, DEM, annual mean temperature (BIO1), precipitation of the driest month (BIO14), and temperature seasonality (BIO4) all exhibit highly significant positive correlations with UMEBLF occurrence (*p* < 0.001). Annual precipitation (BIO12) and isothermality (BIO3) also show significant positive correlations (*p* < 0.05). In contrast, diurnal temperature range (BIO2) and TUHCD display significant negative correlations (*p* < 0.001). Overall, most variables have partial correlation coefficients exceeding ±0.2 and are significant at the 95% or 99% confidence levels (Qiao et al., 2015).

**FIGURE 3:**
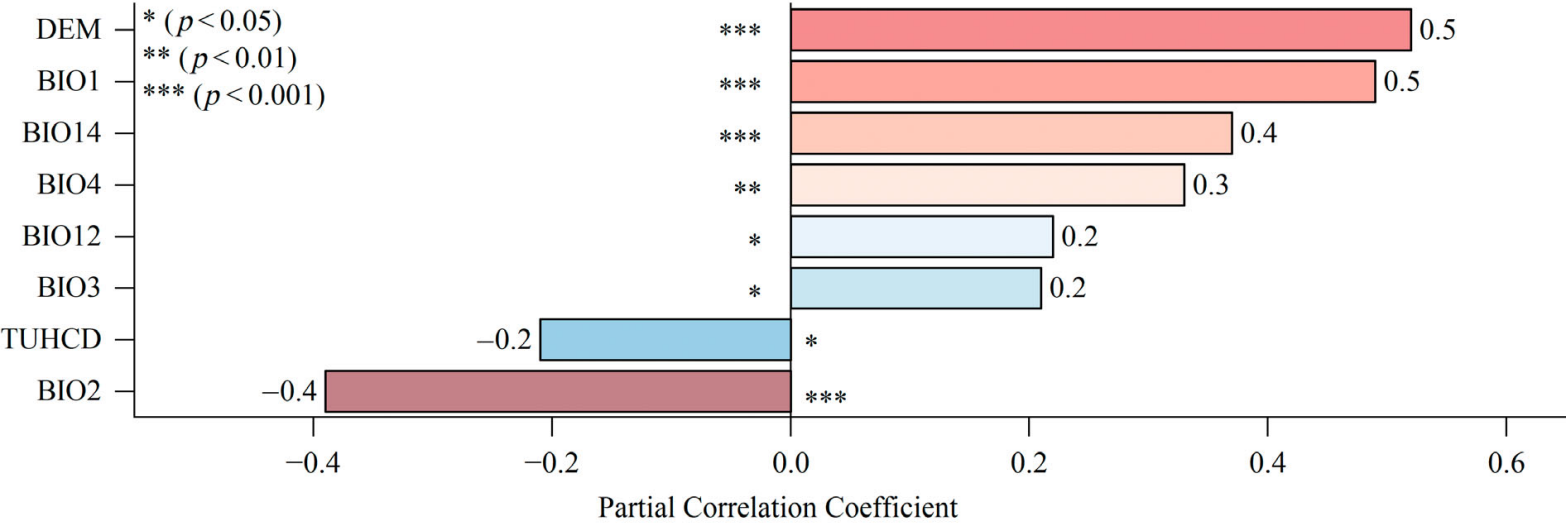
Partial correlation coefficients of the variables. *p < 0.05; **p < 0.01; ***p < 0.001. DEM, digital elevation model; TUHCD, topographic uplift height and cutting depth.

DMTZ, MRAI, and precipitation seasonality (BIO15) were not included in the partial correlation analysis because DMTZ is a categorical variable, whereas MRAI and BIO15 exhibit discontinuous, patch‐like spatial patterns resembling categorical distributions. Previous studies have demonstrated that these three variables have strong ecological relevance for UMEBLF distribution (Ge et al., 2019; Jasiewicz & Stepinski, 2013; Schwager & Berg, 2021), and their spatial patterns are consistent with observed UMEBLF distribution. Accordingly, they were still included in the model construction.

#### Representativeness analysis of presence samples

The representativeness of the selected 54 UMEBLF presence samples with respect to the key environmental variables in the study area determines the accuracy of subsequent modeling (Austin, 2002; Elith et al., 2006; Elith & Leathwick, 2009). PCA was used to project the target samples into environmental principal component space and to evaluate their representativeness along the major environmental gradients. A total of 10,000 random points generated across Yunnan Province were used to extract 11 environmental variables. After removing NoData and abnormal values, 9914 valid points were retained as background points. Among the 11 environmental variables, DMTZ, as a categorical variable, was not included in the PCA and was used only for geomorphological frequency statistics.

PCA of the background points showed that the first two principal components explained 58.70% of the total environmental variation, with the first principal component (PC1) and the second principal component (PC2) accounting for 38.95% and 19.75%, respectively. PC1 mainly reflected integrated environmental information jointly controlled by thermal conditions, elevation, and precipitation. PC2 mainly represented the gradient of environmental variation jointly controlled by mean diurnal range, isothermality, and terrain relief. In the environmental principal component space defined by PC1 and PC2, the 54 UMEBLF presence samples were generally distributed within the core area and showed a certain degree of expansion along both PC1 and PC2, as shown in Figure 4a. This indicates that the presence samples were not restricted to a narrow range of environmental conditions but instead covered the major environmental gradients associated with UMEBLF suitable habitats (Elith et al., 2006; Elith & Leathwick, 2009).

**FIGURE 4:**
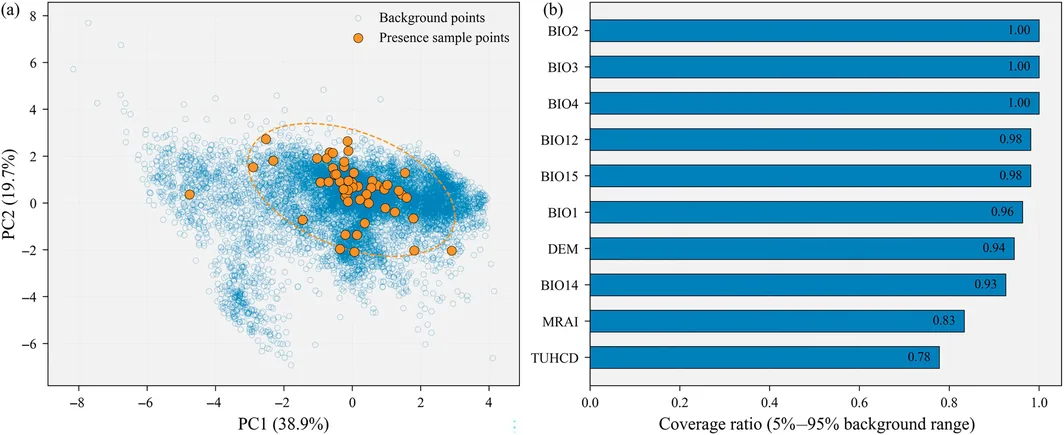
Representativeness of upper montane evergreen broad‐leaved forest (UMEBLF) presence samples in environmental space. (a) Principal components analysis (PCA) environmental space constructed based on continuous climatic variables, topographic variables, and relative topographic position variables. (b) Coverage ratio of UMEBLF presence samples within the 5%–95% main environmental range of the background points. AUC, area under the receiver operating characteristic curve; CatBoost, CatBoost classifier; ExtraTrees, Extremely Randomized Trees; HGB, Histogram‐Based Gradient Boosting; LightGBM, Light Gradient Boosting Machine; RF, Random Forest; TSS, true skill statistic; XGBoost, Extreme Gradient Boosting.

Furthermore, the 5%–95% quantile range of each environmental variable for the background points was defined as the main environmental range of the study area, and the proportion of UMEBLF presence samples falling within this range was calculated to quantify the coverage of the main background environmental gradients by the presence samples, as shown in Figure 4b. The results showed that more than 90% of the UMEBLF presence samples fell within the main environmental range of most climatic variables and the elevation variable of the background points. Specifically, for mean diurnal range (BIO2), isothermality (BIO3), and temperature seasonality (BIO4), 100% of the UMEBLF presence samples fell within the 5%–95% quantile range of the corresponding background variables. For annual mean temperature (BIO1), annual precipitation (BIO12), precipitation seasonality (BIO15), and DEM, the corresponding proportions were 96.30%, 98.15%, 98.15%, and 94.44%, respectively. Because UMEBLF exhibits a certain selectivity for relative mountain position, the coverage proportions of the presence samples for TUHCD and MRAI were 77.78% and 83.33%, respectively. Overall, although the number of presence samples was limited to 54, they adequately represented the major environmental gradient information in the study area (Austin, 2002; Barbet‐Massin et al., 2012; Elith & Leathwick, 2009).

#### Optimal model construction and selection

Machine learning analyses were conducted in Python 3.12.11 using scikit‐learn (Pedregosa et al., 2011), XGBoost (Chen & Guestrin, 2016), LightGBM (Ke et al., 2017), and CatBoost (Prokhorenkova et al., 2018). Six mainstream models were developed to predict habitat suitability probability: RF, Extremely Randomized Trees (ExtraTrees), Extreme Gradient Boosting (XGBoost), Light Gradient Boosting Machine (LightGBM), CatBoost classifier (CatBoost), and Histogram‐Based Gradient Boosting (HGB). After hyperparameter optimization using fivefold cross‐validation (KFold) and grid search (GridSearchCV), all six models achieved AUC values exceeding 0.83 (Figure 5). When evaluated using five metrics, namely, AUC Score, TSS Score, F1 score, AUC CV, and TSS CV, the RF model exhibited the best performance in terms of both accuracy and stability.

**FIGURE 5:**
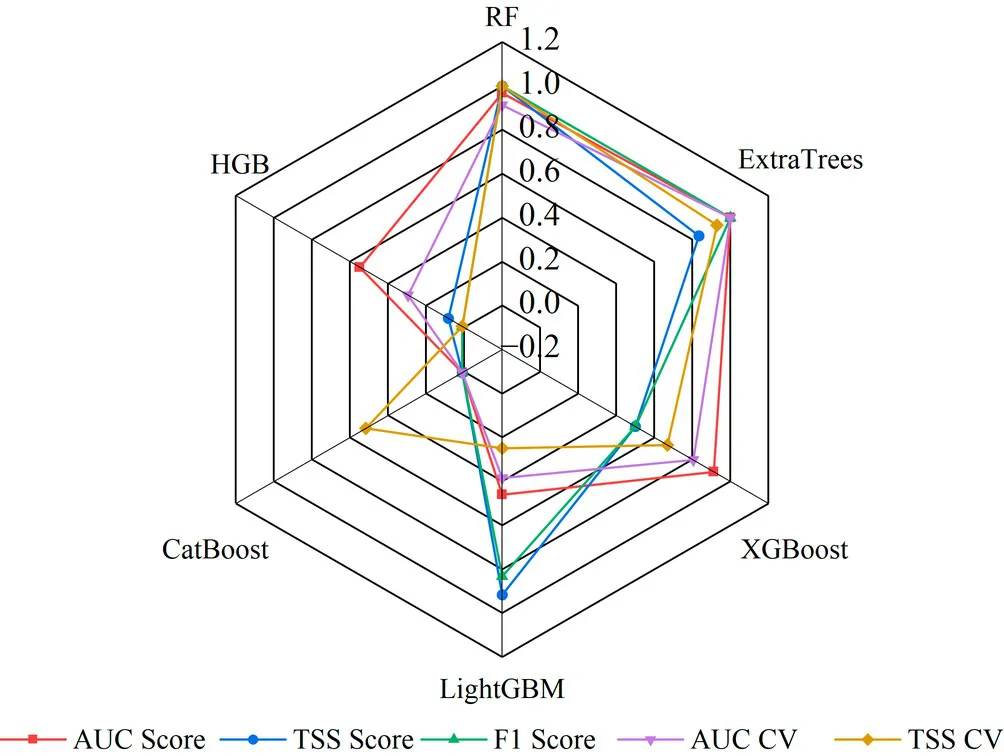
Radar chart comparing the performance of different models across evaluation metrics. AUC, area under the receiver operating characteristic curve; DEM, digital elevation model; DMTZ, digital morphological terrain type.

The final RF parameters were set as follows: *n*_estimators/iterations = 300, max_depth/depth = 5, max_features = “sqrt,” min_samples_split = 5, and min_samples_leaf = 3. After optimization, the RF model achieved an AUC of 0.98, a TSS score of 0.66, and an F1 score of 0.82, while AUC CV and TSS CV were limited to 0.02 and 0.06, respectively.

Statistical validation based on test samples indicated that the RF model provides reliable predictions. As shown in Figure 6a, the F1 score for predicting presence (“1”) reached 0.82, with precision and recall values of 0.74 and 0.93, respectively, indicating strong capability to identify positive samples. For absence (“0”), precision and recall were 0.93 and 0.72, respectively, reflecting a low false‐positive rate. The confusion matrix (Figure 6b) showed that 14 of 15 presence samples were correctly classified, while only one was misclassified as absence. Among absence samples, 13 were correctly classified and 5 were misclassified as presence. The receiver operating characteristic curve (Figure 6c) yields an AUC of 0.93, further confirming the reliability of model predictions.

**FIGURE 6:**
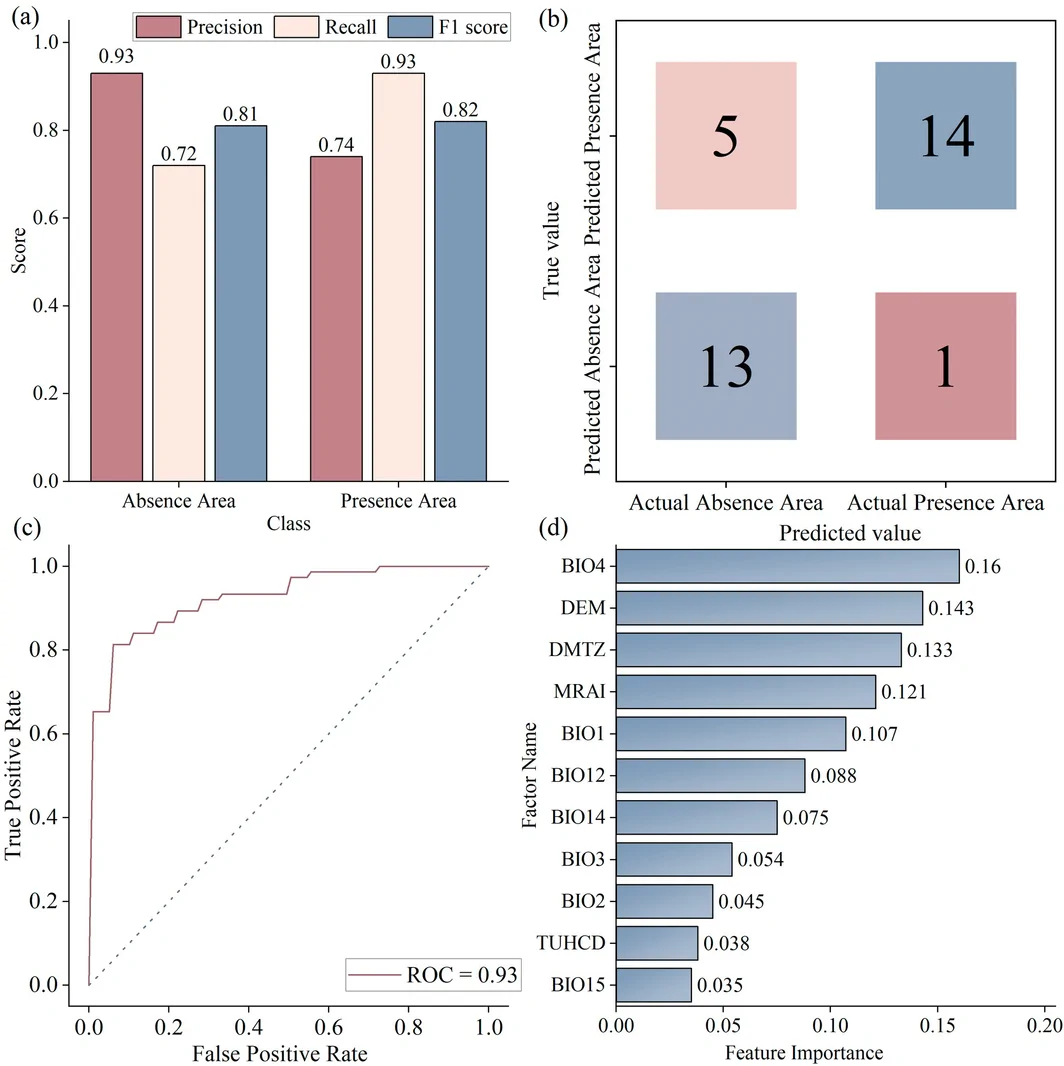
Evaluation of prediction performance of the Random Forest model: (a) Bar chart of evaluation metrics; (b) Confusion matrix; (c) receiver operating characteristic (ROC) curve; (d) Distribution of feature importance. DEM, digital elevation model; DMTZ, digital morphological terrain type; MRAI, mountain relative altitude index; TUHCD, topographic uplift height and cutting depth.

Gini importance was used to evaluate the contribution of training variables in the RF model. The results showed that both topographic and climatic variables contributed substantially to model performance, consistent with the known thermal and moisture requirements of UMEBLF (Tang, 2010; Yang et al., 2025; Zhu, 2021). Overall, the RF model demonstrates good ecological interpretability. As shown in Figure 6d, temperature seasonality (BIO4) exhibited the highest importance, indicating that interannual thermal variability is an important factor influencing UMEBLF habitat suitability. DEM and DMTZ ranked next, suggesting that topography and geomorphology also impose strong constraints. UMEBLF tends to occur in montane areas at middle to high elevations. In addition, annual mean temperature (BIO1), annual precipitation (BIO12), precipitation of the driest month (BIO14), and MRAI also showed notable importance in the model. Furthermore, statistical indicators of suitability probability under current climate conditions across different variable intervals, including the mean, maximum, minimum, and standard deviation, were used to further elucidate the influence of these variables within the RF model.

#### Model robustness assessment

To further examine the influence of sample variation on model performance, repeated subsample modeling was used to assess model robustness (Austin, 2002; Elith & Leathwick, 2009). For each subsampling level of 60%, 70%, 80%, and 90%, 100 subsample sets were randomly generated without replacement from the 54 presence samples and used as presence samples for model construction, corresponding to sample sizes of 32, 38, 43, and 49, respectively. To avoid the uncertainty introduced by the random generation of pseudo‐absence points, a fixed pseudo‐absence set was used in all repeated modeling runs (Zbinden et al., 2024). The RF model parameters in repeated modeling were kept consistent with those of the optimal RF model, including n_estimators = 300, max_depth = 5, max_features = sqrt, min_samples_split = 5, and min_samples_leaf = 3. To reduce the influence of changes in class proportions under different subsampling conditions, the parameter class_weight = balanced was used.

The repeated subsample modeling results showed that the RF model maintained relatively stable predictive performance across different presence‐sample subsampling levels, as shown in Figure 7a. The mean AUC values at the 60%, 70%, 80%, and 90% subsampling levels were 0.887, 0.886, 0.890, and 0.887, with standard deviations of 0.067, 0.063, 0.065, and 0.056, respectively, indicating stable predictive ability under different subsampling conditions. The mean F1 scores were 0.747, 0.782, 0.776, and 0.768, respectively, suggesting that the model's ability to identify presence samples did not decline markedly with changes in the proportion of presence points. The mean TSS values were 0.609, 0.636, 0.627, and 0.602, respectively, indicating that the model's ability to distinguish between the two classes did not show a significant downward trend. These results suggest that the optimal RF model was not sensitive to variations in the presence samples and exhibited good robustness (Breiman, 2001; Elith & Leathwick, 2009).

**FIGURE 7:**
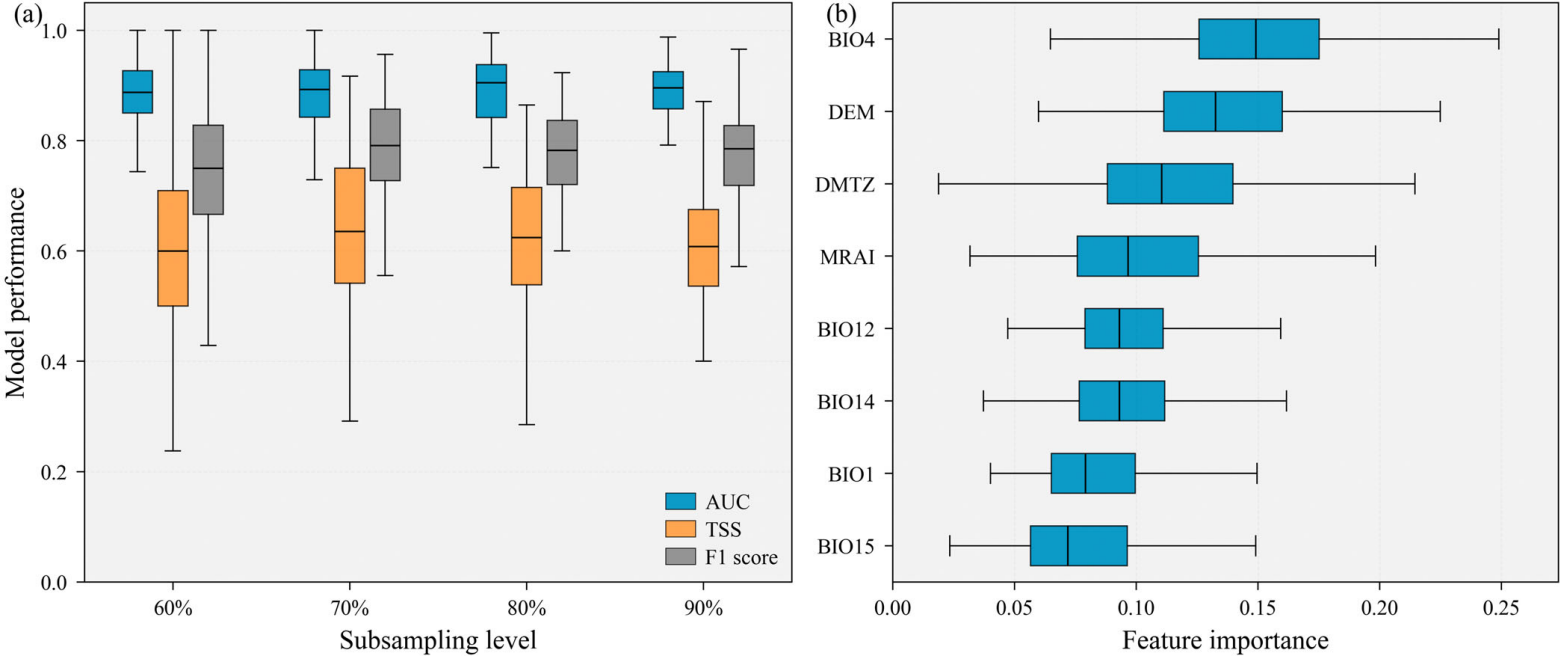
Stability of Random Forest (RF) model performance and environmental variable importance under different subsampling conditions. (a) Distributions of area under the receiver operating characteristic curve (AUC), true skill statistic (TSS), and F1 score obtained from 100 repeated RF modeling runs when the presence samples were subsampled at 60%, 70%, 80%, and 90%, respectively; (b) importance distributions of the top eight environmental variables ranked by mean importance across repeated modeling runs. The upper and lower boundaries of each box represent the 75th and 25th percentiles, respectively; the central line represents the median; and the whiskers represent the range of non‐outlier values. DEM, digital elevation model; MRAI, mountain relative altitude index; RF, Random Forest; TUHCD, topographic uplift height and cutting depth.

Variable importance analysis further showed that major environmental variables, including temperature seasonality (BIO4), DEM, DMTZ, and MRAI, maintained stable explanatory effects under different subsampling conditions, as shown in Figure 7b. Among them, temperature seasonality (BIO4) and DEM consistently showed relatively high importance values across all subsampling levels. DMTZ, MRAI, annual precipitation (BIO12), precipitation of the driest month (BIO14), annual mean temperature (BIO1), and precipitation seasonality (BIO15) also made stable contributions. For example, the mean importance of temperature seasonality (BIO4) ranged from 0.147 to 0.156 across the 60%–90% subsampling levels; that of DEM ranged from 0.133 to 0.140; that of DMTZ ranged from 0.109 to 0.121; and that of MRAI ranged from 0.100 to 0.103. These comparisons indicate that the identification of environmental drivers of UMEBLF habitat suitability did not rely strongly on any single sample partition, suggesting that the samples had good representativeness.

### Suitable habitat mapping

Using the optimized RF model, suitability probabilities were calculated for each pixel under current climatic conditions (1970–2000) and future scenarios (SSP1‐2.6, SSP3‐7.0, and SSP5‐8.5). These values were used for predictive simulation and mapping (Guisan et al., 2017). The results were generated in raster format and classified into four suitability levels using the Natural Breaks method (Tang et al., 2021): Optimally Suitable, Highly Suitable, Moderately Suitable, and Marginally Suitable. The cloud–fog belt boundary was used solely as an independent ecological reference. Spatial overlay and visual interpretation were conducted to assess whether predicted Optimally Suitable areas under current climate conditions were consistent with observed climatic patterns.

### Conservation analysis

Optimally Suitable areas under both current and future climate scenarios were overlaid with nature reserve boundaries to quantify the area and proportion of Optimally Suitable habitat within protected areas. This analysis was used to identify potential conservation gaps where theoretically optimal UMEBLF habitat remains unprotected. Prior to overlay analysis, raster outputs of Optimally Suitable areas were converted to vector format. Processing steps included removal of fragmented polygons smaller than 20 km^2^, polygon boundary smoothing using a tolerance parameter of 25 km, and spatial join and clipping operations with nature reserve boundaries.

## RESULTS

### Suitable distribution of UMEBLF under current and future conditions

#### Suitable habitat distribution under current climate conditions

Under current climate conditions (1970–2000), areas with suitable habitat for UMEBLF are primarily concentrated in the mid‐montane regions of central and western Yunnan, forming a distinct banded pattern aligned with major mountain ranges (Figure 8). Spatially, this forest type is strongly controlled by northwest–southeast‐oriented longitudinal mountain systems. Along its north–south extent, the suitable belt is intermittently segmented by secondary ridges and valleys, resulting in locally fragmented strip‐like or island‐like patterns.

**FIGURE 8:**
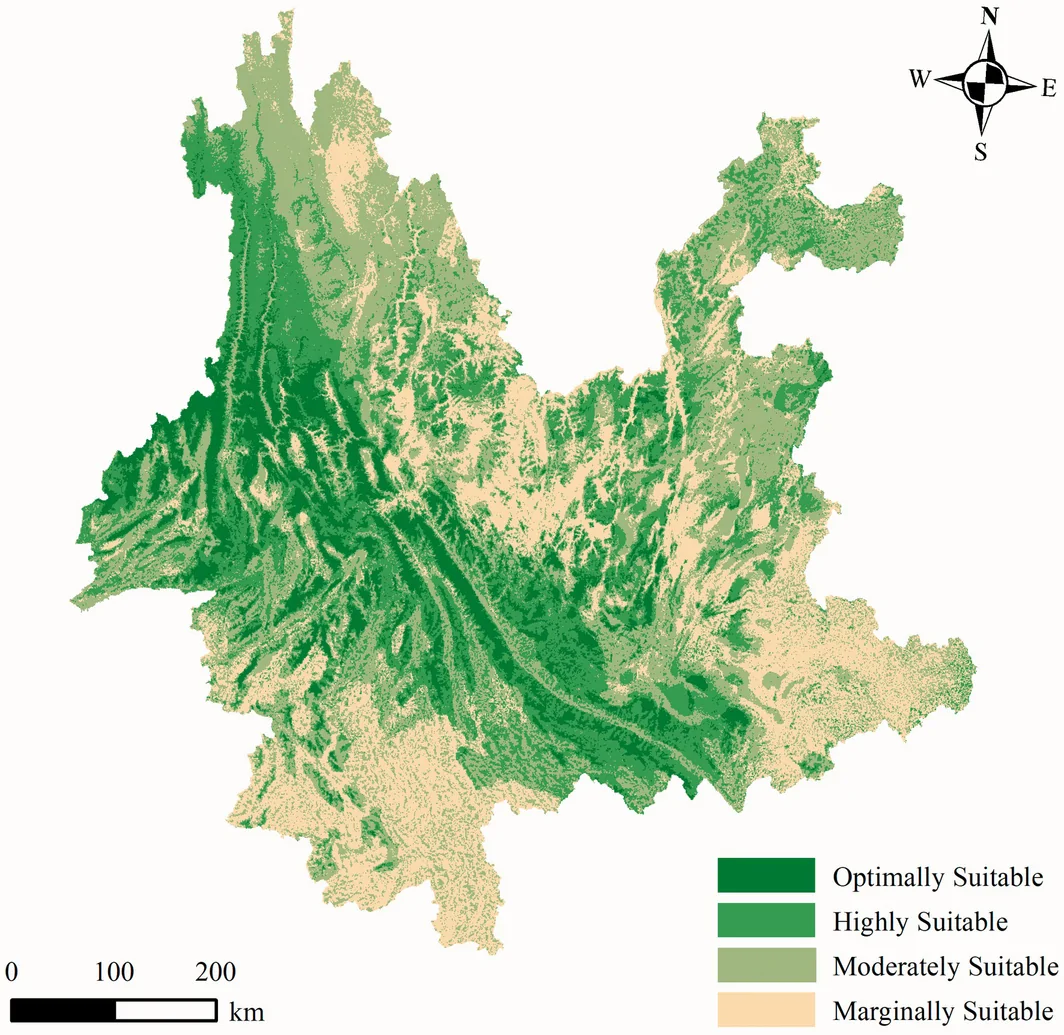
Distribution of suitable habitat areas in Yunnan Province under current climate conditions.

Optimally Suitable areas are mainly distributed across mountain ranges such as the Ailao, Wuliang, Gaoligong, Yunling, and Nu Mountains, with some areas extending into mid‐elevation mountains of northwestern and southern Yunnan. In contrast, Marginally Suitable areas are widely distributed in low‐elevation basins, plains, and major river valleys. Overall, the spatial pattern of UMEBLF is characterized by greater suitability in western than eastern areas, in southern than northern regions, and in mountainous rather than in basins.

Area statistics indicate that Optimally Suitable habitat covers approximately 4.2 × 10^4^ km^2^, accounting for 10.6% of the study area. Highly Suitable habitat occupies 10.7 × 10^4^ km^2^ (27.3%), Moderately Suitable habitat covers 14.9 × 10^4^ km^2^ (37.9%), and Marginally Suitable habitat covers 9.6 × 10^4^ km^2^ (24.3%).

#### Validation of optimally suitable areas under current climate conditions

UMEBLF distribution is closely associated with specific thermal and moisture conditions, and Optimally Suitable areas exhibit distinct local climatic characteristics. A clear spatial correspondence can be observed between current Optimally Suitable areas and the cloud–fog belt (Figure 9). Both display banded spatial patterns that generally follow the orientation of mountains and valleys, extending from northwest to southeast and running approximately parallel to contour lines.

**FIGURE 9:**
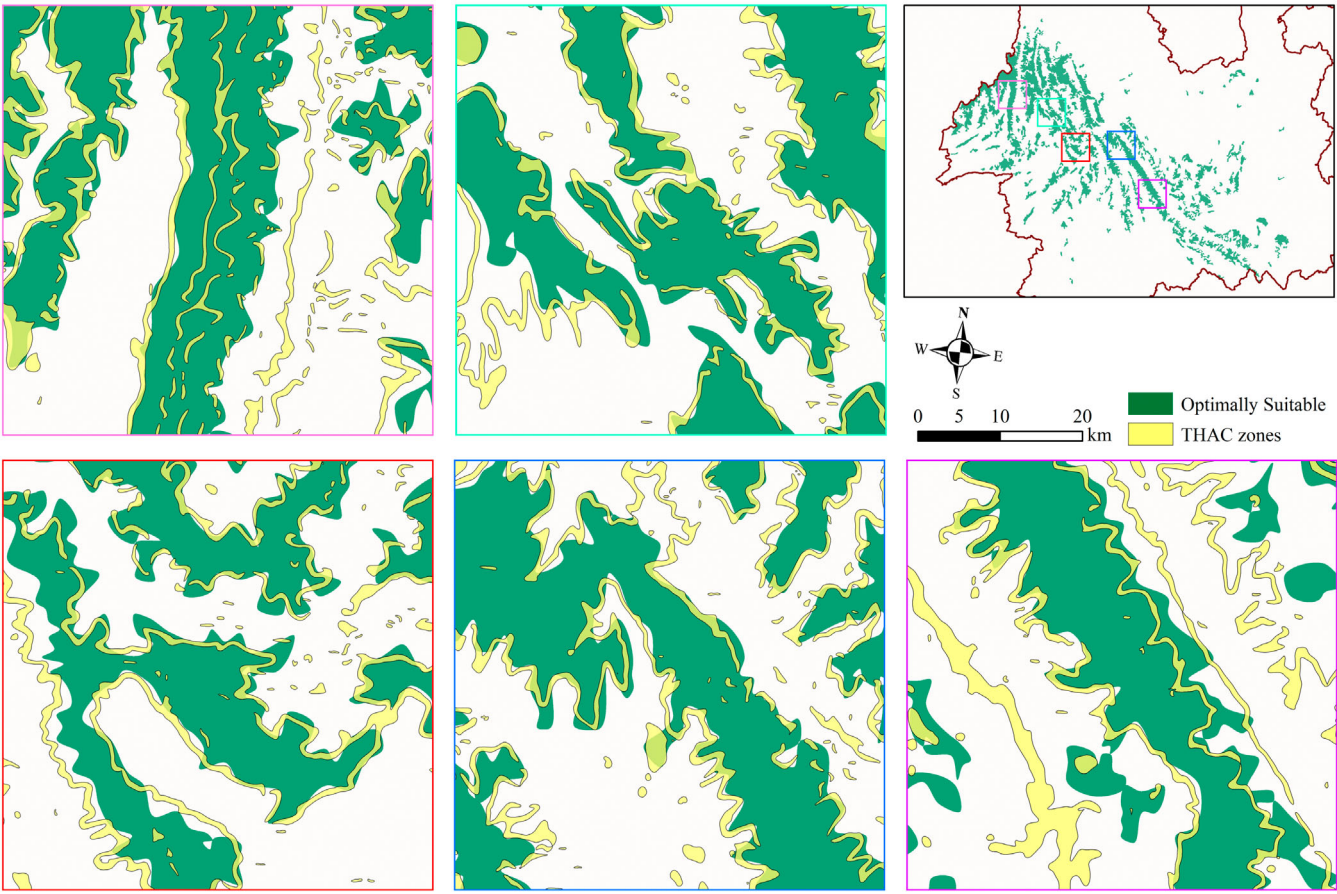
Overlay of Optimally Suitable areas of upper montane evergreen broad‐leaved forest (UMEBLF) and the cloud–fog belt in Yunnan Province under current climate conditions. Transient humid altitudinal cloud (THAC) zones refer to the cloud–fog belt areas.

Optimally Suitable areas are predominantly located on middle and upper slopes, typically at elevations corresponding to approximately two‐thirds or more of the mountain height above the base. In the Cangshan–Ailao Mountain region, the lower boundary of Optimally Suitable habitat occurs at higher elevations on windward slopes than on leeward slopes. On the western slope of the Gaoligong Mountains, this lower boundary can descend below 1500 m and overlap with the cloud–fog belt near the mountain foot, whereas on the eastern slope, it generally corresponds with the cloud–fog belt boundary only on middle and upper slopes between 1500 and 2000 m.

The ecological and climatic characteristics of the montane cloud–fog belt create stable thermal and moisture conditions on upper slopes that are highly favorable for UMEBLF growth. Previous studies indicate that within the cloud–fog belt, surface temperature can increase by an average of 6.32°C per 100‐m decrease in elevation, substantially exceeding the normal rate (0.65°C per 100 m; Wang et al., 2024). Over short elevational gradients, annual mean relative humidity can rise from below 60% to above 87% and remain more than 10% higher than humidity levels at the 600‐hPa atmospheric layer above. These abundant heat and moisture conditions create a climatic environment well suited to UMEBLF growth.

#### Suitable distribution under future climate scenarios

Under future climate change, the overall spatial distribution of suitable UMEBLF habitat exhibits a contraction in Optimally Suitable areas and expansion of Marginally Suitable areas (Figure 10). Under low‐emission scenarios such as SSP1‐2.6, changes in suitability are relatively minor. In contrast, under medium‐ (SSP3‐7.0) and high‐emission (SSP5‐8.5) scenarios, suitability degradation becomes more pronounced, with Highly Suitable areas gradually shifting to Moderately Suitable and Marginally Suitable categories.

**FIGURE 10:**
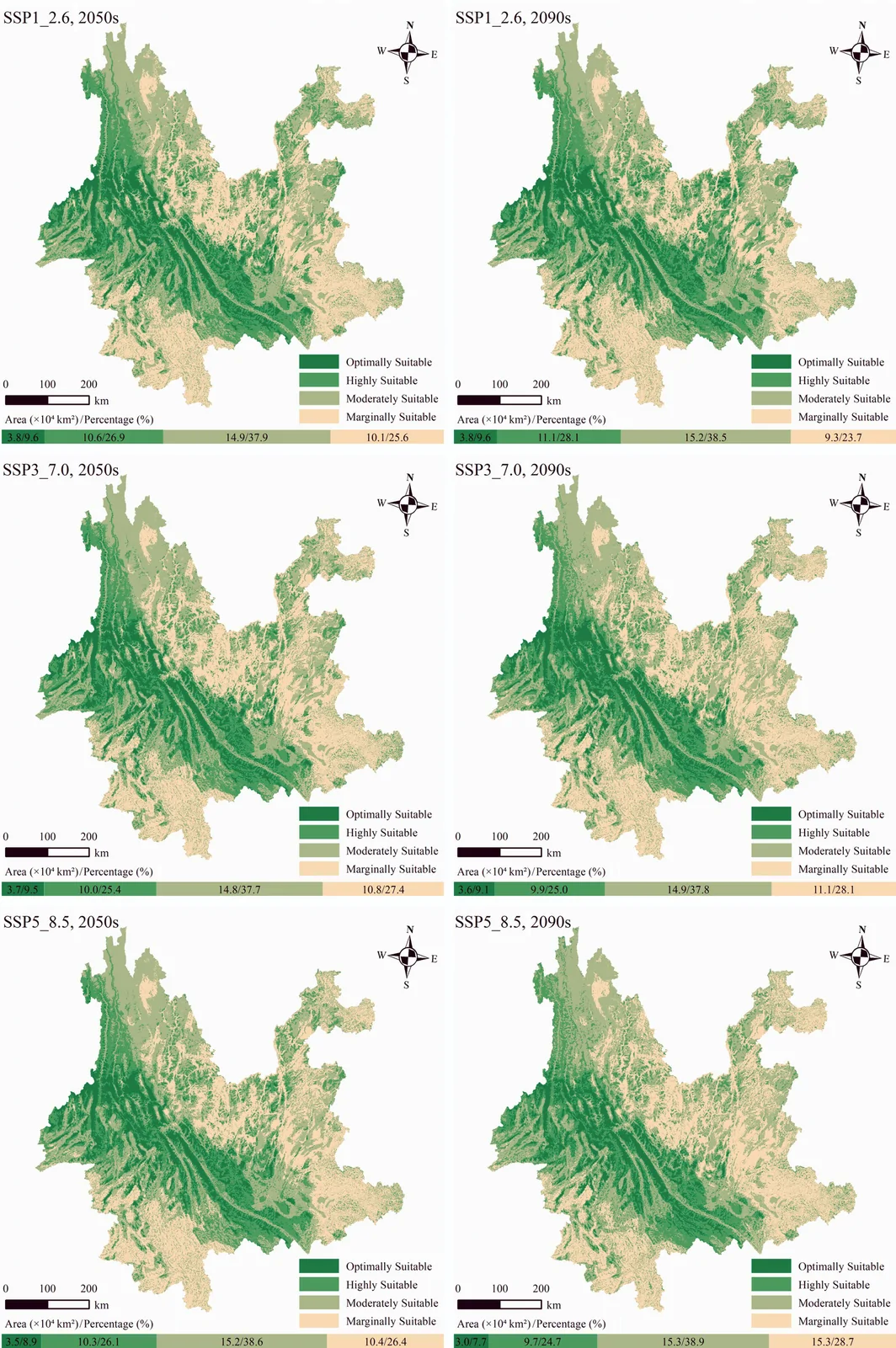
Distribution of suitable habitat areas in Yunnan Province under future climate scenarios, with area and percentage statistics.

At present, Optimally Suitable habitat accounts for 10.6% of the study area (approximately 4.2 × 10^4^ km^2^). Under SSP1‐2.6, this proportion decreases slightly to 9.6%, with approximately 3.8 × 10^4^ km^2^ of Optimally Suitable habitat remaining by the 2090s. The proportion of Highly Suitable habitat fluctuates slightly and reaches 28.1% in the 2090s, indicating that this forest type retains relatively strong climate adaptability under low‐emission pathways. Moderately Suitable and Marginally Suitable areas remain relatively stable at approximately 38% and 24%, respectively.

In contrast, under SSP3‐7.0 and SSP5‐8.5, Highly Suitable habitat contracts substantially, and Optimally Suitable habitat declines to 9.1% (3.6 × 10^4^ km^2^) and 7.7% (3.0 × 10^4^ km^2^), respectively. Meanwhile, Moderately Suitable habitat expands markedly, reaching 38.9% (over 15.3 × 10^4^ km^2^) by the 2090s under SSP5‐8.5. These trends indicate that higher carbon emissions are associated with stronger suppression of Optimally Suitable and Highly Suitable habitats, while Marginally Suitable areas continue to expand, reflecting an overall shift of habitat suitability toward lower levels.

### Conservation of optimally suitable areas of UMEBLF

#### Current protection status

Overlay analysis reveals a high degree of spatial correspondence between current Optimally Suitable areas and nature reserve boundaries (Figure 11). In both central and western Yunnan, nature reserves largely coincide with suitable UMEBLF habitat. The boundaries of Optimally Suitable areas generally align with those of existing nature reserves, indicating effective spatial alignment between suitable habitats and current conservation areas.

**FIGURE 11:**
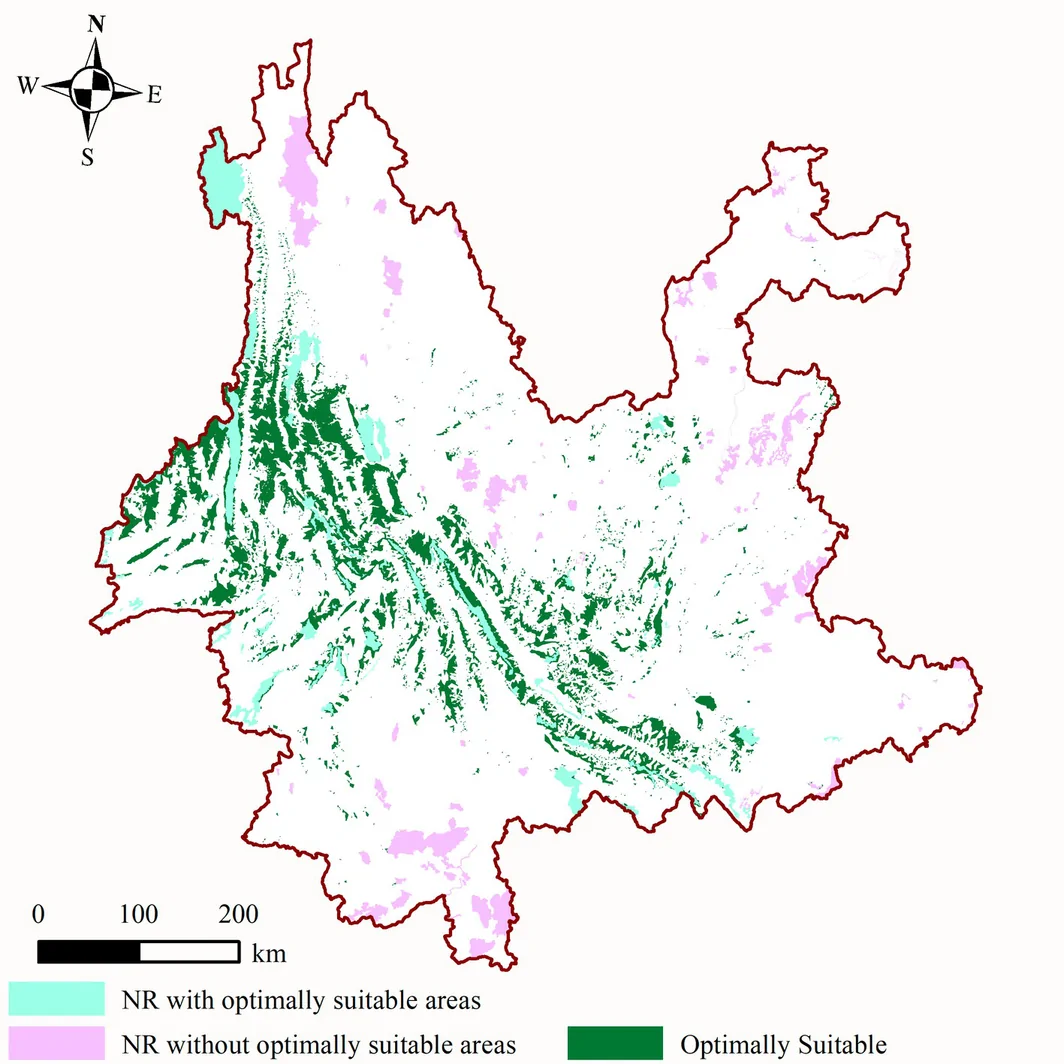
Overlay of Optimally Suitable habitat patches and nature reserve boundaries in Yunnan Province under current climate conditions. NR, nature reserves.

A total of 32 nature reserves contain Optimally Suitable UMEBLF habitat, of which 21 are at the provincial level or higher. Within these reserves, Optimally Suitable habitat covers 5655 km^2^ (20.3% of total reserve area), Highly Suitable habitat covers 7247 km^2^ (26.0%), Moderately Suitable habitat covers 9901 km^2^ (35.6%), and Marginally Suitable habitat covers 4997 km^2^ (18.0%).

Approximately 75% of these reserves are located on the southwestern side of the Cangshan–Ailao Mountain region. Reserves where Optimally Suitable habitat exceeds 80% of total reserve area include Wuliangshan National Nature Reserve, Ailaoshan National Nature Reserve, Yuanyang Guanyinshan Provincial Nature Reserve, Lincang Lancangjiang Provincial Nature Reserve, and Yongping Jinguangsi Provincial Nature Reserve, with corresponding areas of 332, 644, 139, 754, and 76 km^2^. Notably, the national reserves in the Wuliangshan and Ailaoshan Mountains, which serve as important barriers to warm and humid airflows, are almost entirely occupied by Optimally Suitable habitat.

Approximately 25% of reserves are situated on the northeastern side of the Cangshan–Ailao Mountain line. In this region, Wenshan National Nature Reserve contains 191 km^2^ of Optimally Suitable UMEBLF habitat, Jiaozishan National Nature Reserve contains 91 km^2^, and Mopanshan County‐level Forest Ecological Nature Reserve in Xinping County contains 68 km^2^.

In the Gaoligongshan National Nature Reserve, 27% of the area (1173 km^2^) is classified as Highly Suitable for UMEBLF. Other reserves with substantial Optimally Suitable habitat include Jinping Watershed National Nature Reserve, Nangunhe National Nature Reserve, and Tongbiguan National Nature Reserve, with areas of 208, 142, and 119 km^2^, accounting for 47%, 27%, and 23% of their total areas, respectively. Daweishan National Nature Reserve contains a smaller Optimally Suitable area of 27 km^2^ (6%). In this region, UMEBLF is mainly distributed along upper mountainous watersheds between major rivers such as the Honghe, Lancangjiang, and Nujiang at elevations of approximately 2000–2700 m.

#### Conservation analysis of UMEBLF under future climate scenarios

##### (1) Changes across the entire region

Under future climate scenarios, the proportions of different habitat suitability levels within nature reserves change markedly, with Optimally Suitable areas gradually shrinking, Highly Suitable and Moderately Suitable areas fluctuating, and Marginally Suitable areas expanding (Figure 12).

**FIGURE 12:**
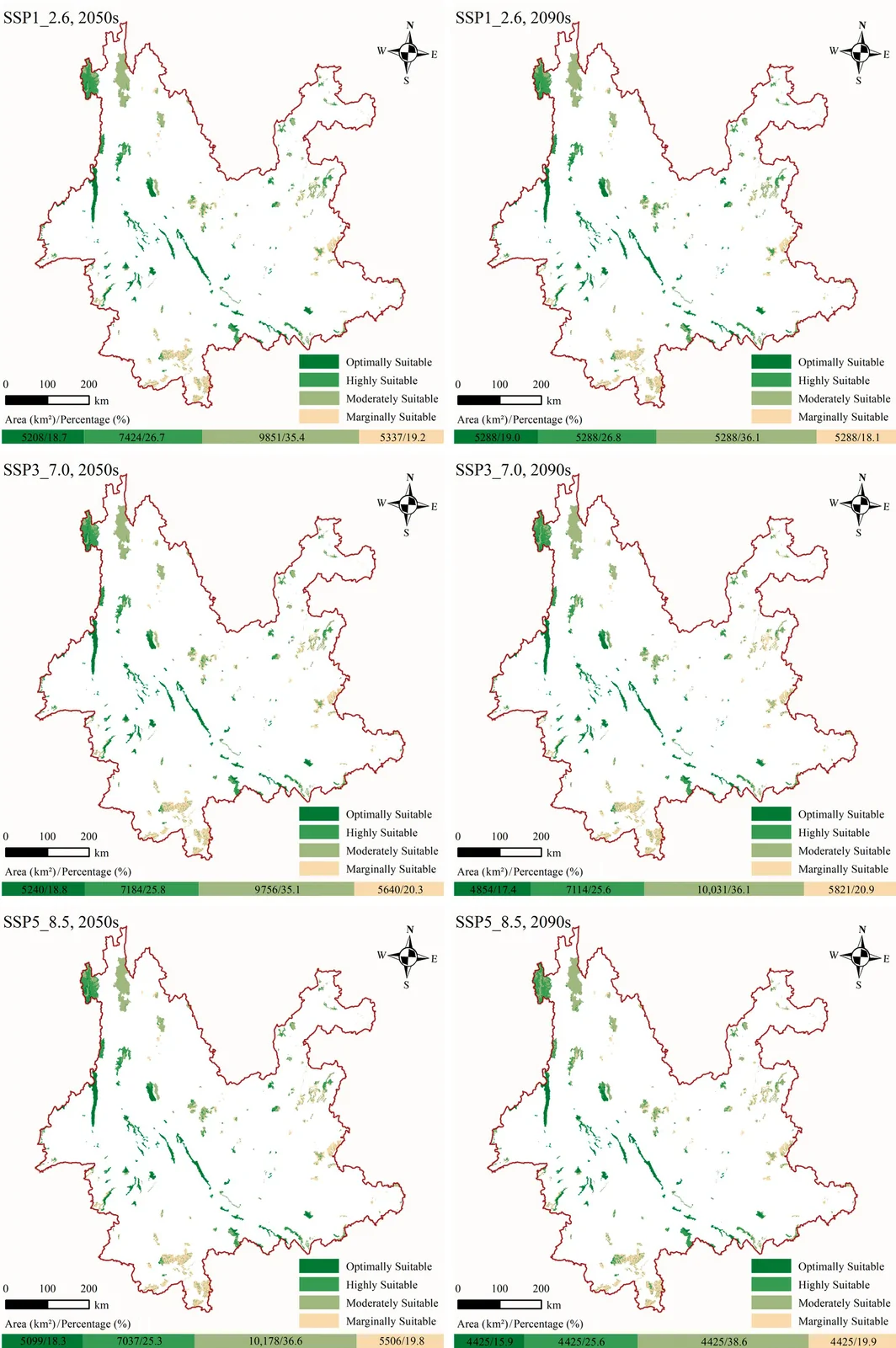
Distribution of suitable habitat areas within nature reserves in Yunnan Province under future climate scenarios, with area and percentage statistics.

Relative to current conditions, under SSP1‐2.6 the Optimally Suitable area decreases to 5208 km^2^ (18.7%) in the 2050s and slightly increases to 5288 km^2^ (19.0%) in the 2090s. Under SSP3‐7.0 and SSP5‐8.5, declines are more pronounced. In SSP3‐7.0, Optimally Suitable habitat decreases to 4854 km^2^ (17.4%) by the 2090s, while under SSP5‐8.5, it further decreases to 4425 km^2^ (15.9%). These results indicate a persistent contraction of high‐quality habitat under increasing emission pressure.

Highly Suitable habitat exhibits relatively minor fluctuations across scenarios, remaining between 7037 and 7447 km^2^ (±2%). Moderately Suitable habitat expands slightly under multiple scenarios, reaching 10,739 km^2^ (38.60%) by the 2090s under SSP5‐8.5, representing the largest increase among all suitability classes. This suggests that while suitability may improve in some marginal regions, the overall habitat quality may decline.

Marginally Suitable habitat also increases, rising to 5821 km^2^ (20.9%) under SSP3‐7.0 and 5541 km^2^ (19.9%) under SSP5‐8.5 by the 2090s. These trends indicate that suitable habitat areas are shifting toward less favorable environmental conditions in some regions.

##### (2) Changes in the case study area

The Gengma section of the Lancang River Provincial Nature Reserve in Lincang, Yunnan Province, was selected as a representative case because UMEBLF is one of its primary conservation targets, and the area is representative in terms of both ecological niche characteristics and projected future changes. The geographic location and elevation distribution of the case‐study area are shown in Figure 13; the spatial distribution, area, and proportion of suitable UMEBLF habitat under current climatic conditions are shown in Figure 14; and the spatial distribution and changes in the area and proportion of suitable UMEBLF habitat under three climate scenarios for two future periods are shown in Figure 15.

**FIGURE 13:**
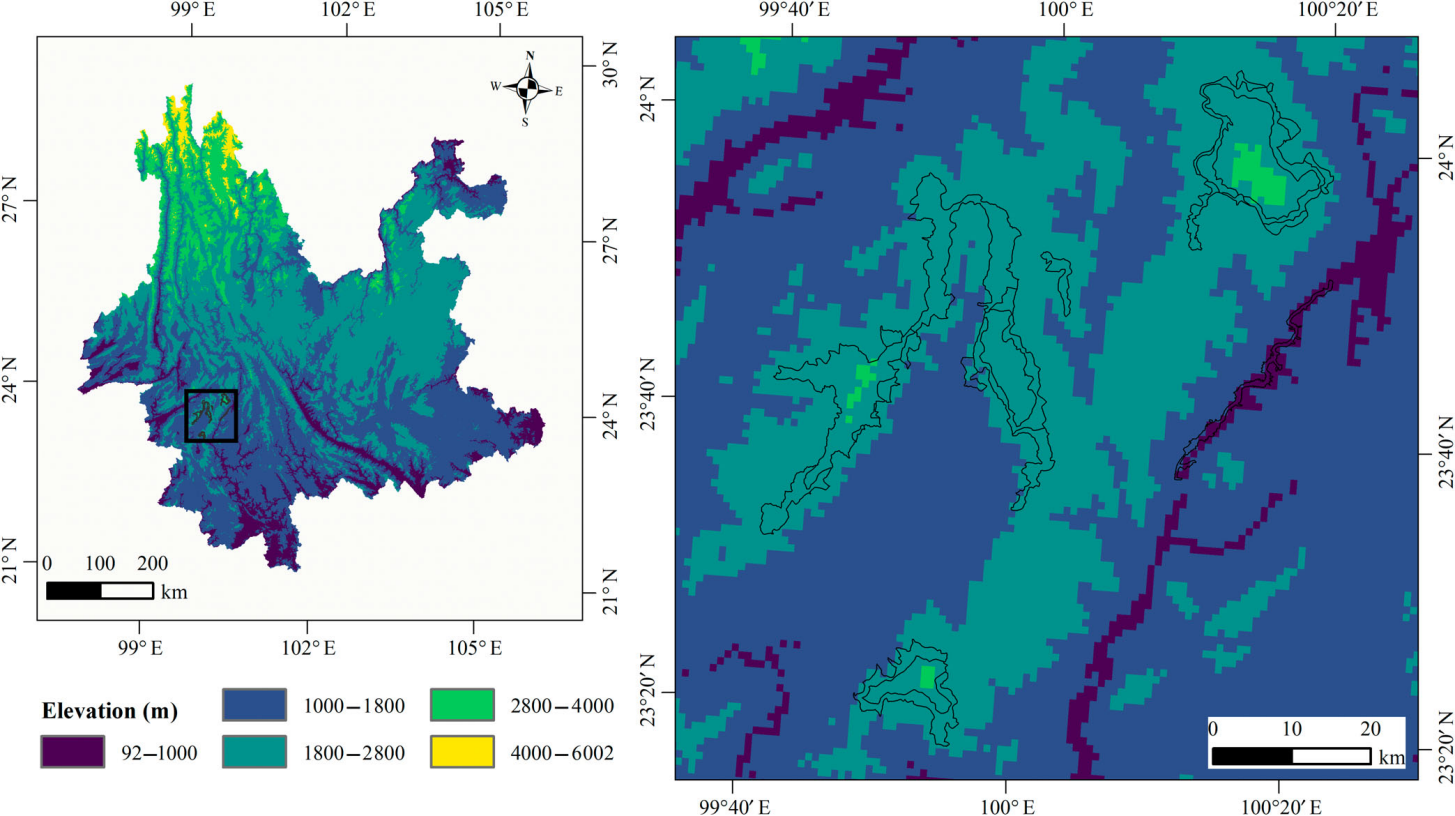
Location and elevation distribution of the Gengma section of the Lancang River Provincial Nature Reserve in Lincang, Yunnan Province.

**FIGURE 14:**
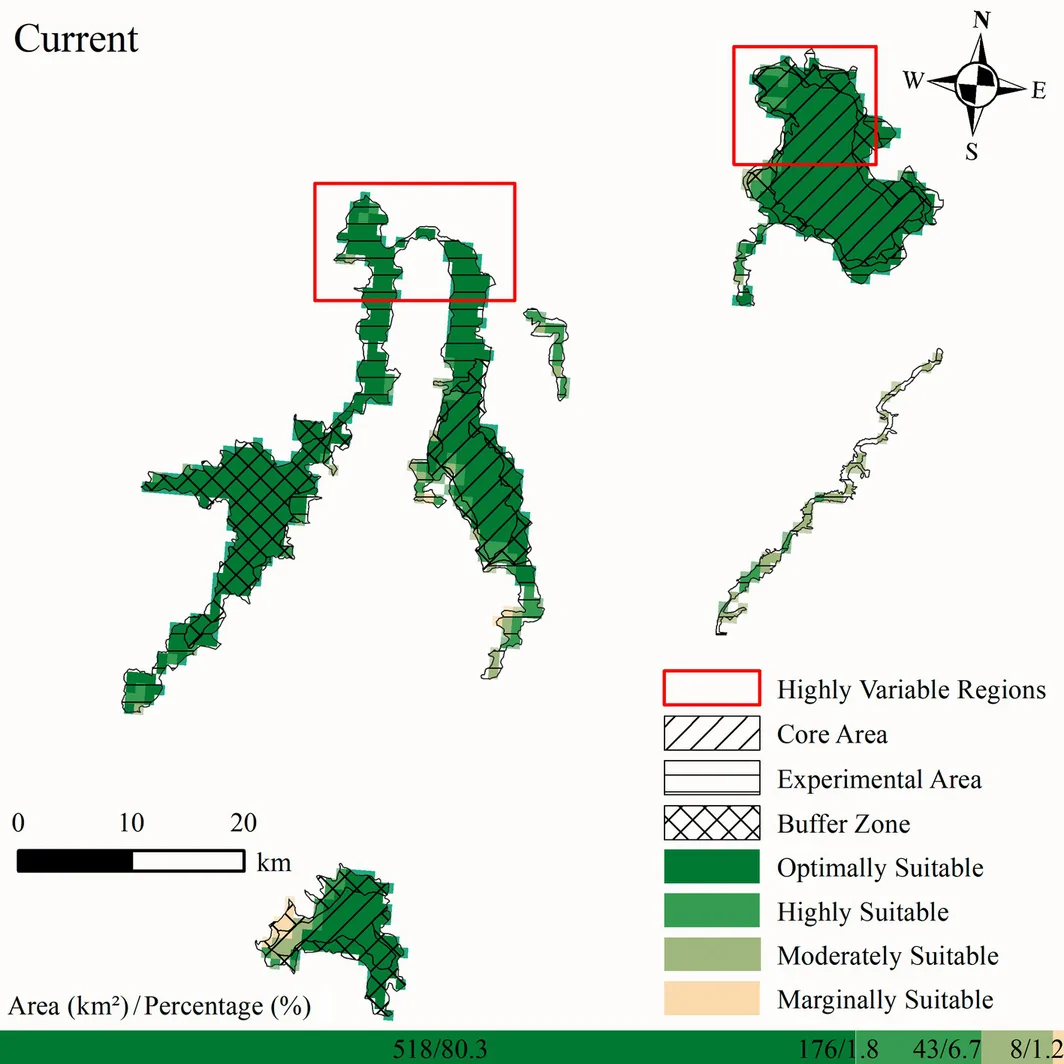
Distribution, area, and proportion of suitable upper montane evergreen broad‐leaved forest (UMEBLF) habitat in the Gengma section of the Lancang River Provincial Nature Reserve under current climatic conditions. The red boxes indicate areas selected for comparison with future climate scenarios.

**FIGURE 15:**
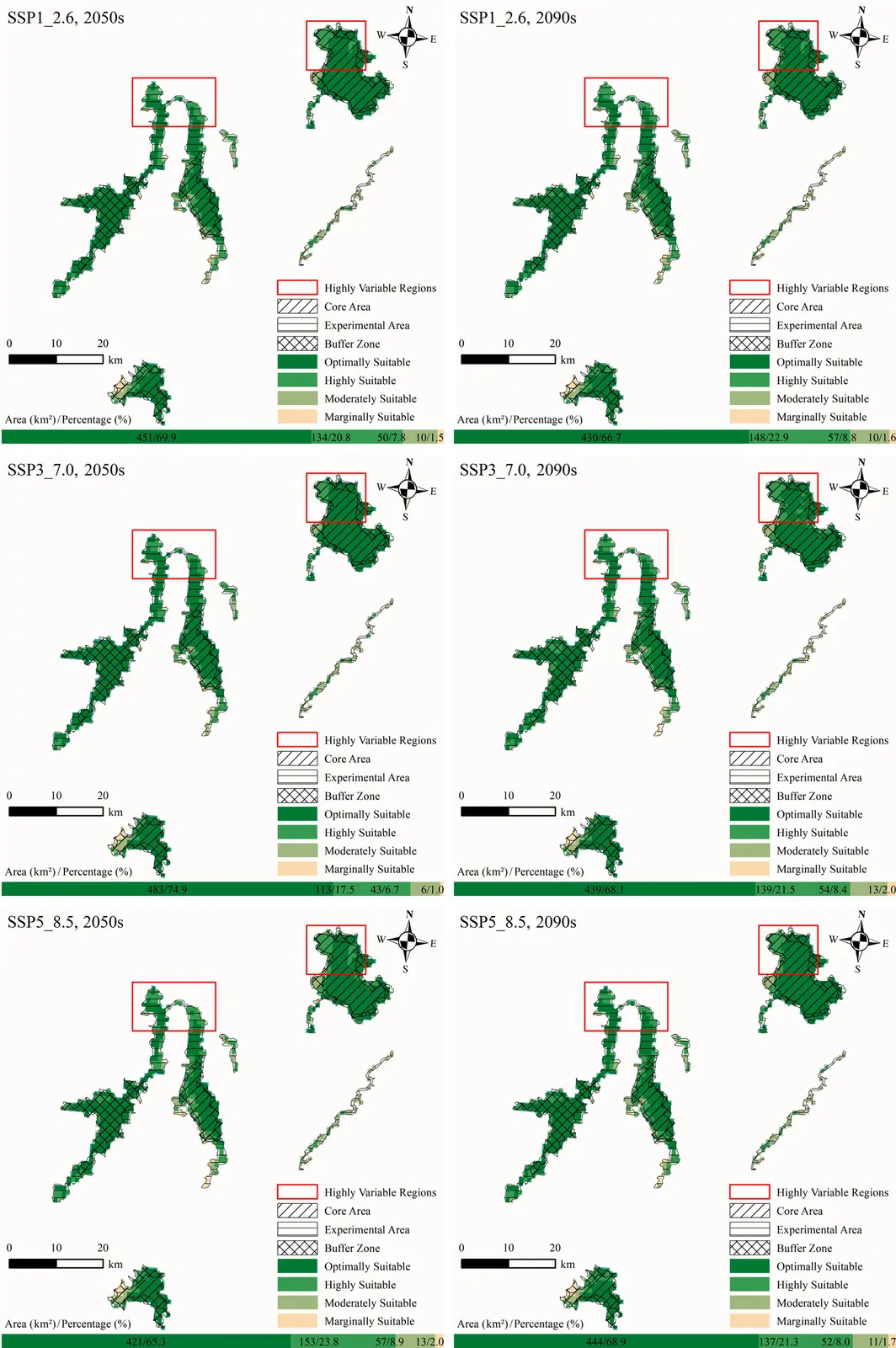
Distribution, area, and proportion of suitable upper montane evergreen broad‐leaved forest (UMEBLF) habitat in the Gengma section of the Lancang River Provincial Nature Reserve under future climate scenarios. The red boxes indicate areas with more pronounced changes.

This area is located within the mid‐montane zone of Yunnan and is strongly influenced by the southwest monsoon. UMEBLF forms a continuous and clearly defined Optimally Suitable belt, reflecting its ecological response to the coupled effects of monsoon dynamics, topography, and thermal and moisture conditions. Model results indicate that this reserve experiences some of the most pronounced suitability transitions among protected areas in Yunnan under future climate scenarios, with changes mainly occurring along its northern boundary.

The reserve includes both core and experimental zones, and projected habitat degradation is concentrated mainly in the experimental zone where limited human activities are permitted. This setting makes the Gengma section particularly suitable for examining management risks arising from the combined effects of climate change and anthropogenic disturbance.

Under future climate scenarios, overall suitability patterns show moderate fluctuations, but Optimally Suitable habitat along marginal areas exhibits a clear degradation trend. Under current conditions, Optimally Suitable habitat is mainly located within the core protection zone. Under SSP1‐2.6, Optimally Suitable area is projected to decrease by 67 km^2^ in the 2050s and 88 km^2^ in the 2090s. Under SSP3‐7.0, reductions of 35 and 44 km^2^ are projected for the 2050s and 2090s, respectively, totaling 79 km^2^. Overall, changes remain relatively limited and are concentrated along the northern margin of the reserve. Under SSP5‐8.5, Optimally Suitable habitat area decreases by 97 km^2^ in the 2050s, the lowest value among all scenarios, and shows a slight recovery in the 2090s, still remaining below current levels.

Meanwhile, under medium‐emission scenarios, Marginally Suitable habitat expands from 8 km^2^ under present conditions to 13 km^2^, primarily along the edges of the reserve. This suggests that suitability for UMEBLF in the peripheral areas, which correspond to the experimental zone, is more sensitive to climate warming. Since limited human activities are permitted in the experimental zone, this trend may further increase the stress risk faced by UMEBLF.

### Influence of environmental factors on the suitable distribution of UMEBLF

#### Thermal conditions

From a thermal perspective (Figure 16a), UMEBLF distribution is not driven by high temperatures alone but is jointly constrained by minimum temperature thresholds and thermal stability. Optimally Suitable areas do not exhibit a pattern in which higher mean annual temperature leads to higher suitability. Instead, a clear minimum temperature threshold is evident. The minimum annual mean temperature within Optimally Suitable areas is 3.94°C, whereas minimum values in other suitability classes fall below 0°C, indicating that low‐temperature stress is a primary factor limiting the spatial distribution of UMEBLF. Under otherwise similar conditions, higher minimum temperature thresholds favor UMEBLF persistence.

**FIGURE 16:**
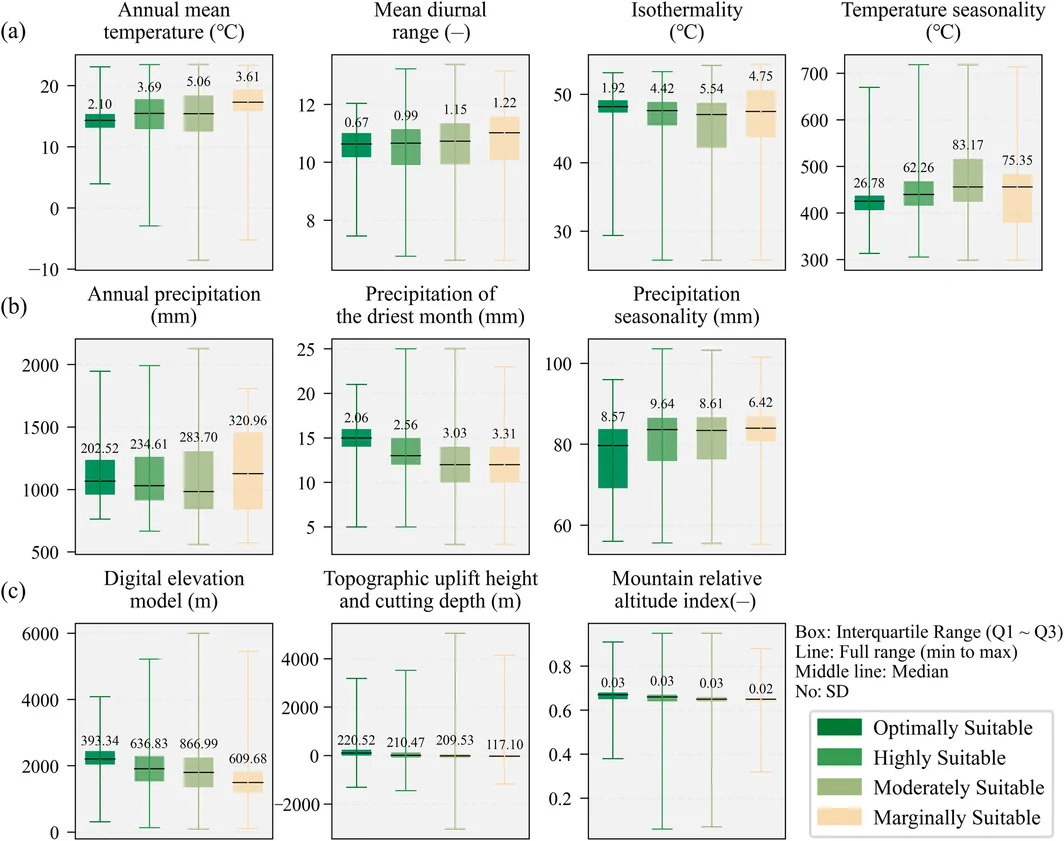
Distribution of environmental variables under different suitability levels. (a) Thermal factors; (b) moisture factors; (c) topographic and geomorphological factors.

Diurnal temperature range (BIO2) and temperature seasonality (BIO4) show higher minimum values and lower variability within Optimally Suitable areas, with minimum values of 7.45°C and 313.40°C, respectively. These values decrease gradually from Optimally Suitable to Marginally Suitable classes, indicating that UMEBLF tends to occur in areas with small temperature fluctuations and greater thermal stability. Isothermality (BIO3) similarly exhibits higher mean and minimum values and the lowest standard deviation in Optimally Suitable areas, further indicating that smaller diurnal temperature differences and greater environmental stability favor the distribution of UMEBLF.

#### Moisture conditions

From a moisture perspective (Figure 16b), Optimally Suitable UMEBLF habitat does not correspond to the highest mean annual precipitation. Although the mean, median, and upper quantiles of annual precipitation (BIO12) are not the highest, Optimally Suitable areas exhibit relatively small standard deviation and higher minimum and maximum values than those of other suitability classes. This indicates that UMEBLF depends more on reliable moisture availability under extreme conditions than on increased average precipitation.

Precipitation during the driest month (BIO14) plays a more direct limiting role. Mean, median, and upper quantiles of BIO14 are substantially higher in Optimally and Highly Suitable areas than in Moderately and Marginally Suitable areas, indicating that the moisture threshold during the dry season is a key factor for maintaining the stability of UMEBLF distribution. In contrast, maximum BIO14 values and standard deviation are not critical limiting factors, suggesting that this forest type is more sensitive to sustained dry‐season moisture rather than isolated extreme events. Precipitation seasonality (BIO15) is also lower in Optimally Suitable areas, indicating that stable intra‐annual moisture regimes favor UMEBLF distribution, consistent with the stability‐driven trend observed for thermal variables.

#### Topographic and geomorphological factors

In terms of continuous topographic variables (Figure 16c), Optimally Suitable UMEBLF areas are generally located at higher elevations and in areas with more pronounced terrain relief. Mean elevation (DEM), TUHCD, and MRAI values are 2242 m, 134 m, and 0.67, respectively, with corresponding 75th percentiles of 2445 m, 246 m, and 0.68. These results indicate that UMEBLF tends to occupy specific slope positions within the mid‐montane zone, particularly middle and upper slopes of elevated mountain bodies that stand above the surrounding TTSA.

Importantly, the newly introduced geoscientific variables TUHCD and MRAI reveal that suitable UMEBLF habitat distribution is not controlled solely by absolute elevation but is closely linked to relative topographic position formed during the blocking, uplift, and energy accumulation of warm and humid air masses. This highlights the dominant role of the coupling mechanism between terrain and thermal and moisture conditions, rather than any single factor acting alone, in shaping the local distribution pattern in monsoon‐influenced mountainous regions.

From the perspective of categorical variables, DMTZ differs significantly among suitability classes (Appendix S1: Table S1). Optimally and Highly Suitable habitats are mainly associated with “mid‐ to high‐elevation mountains with large relief” and “mid‐ to high‐elevation mountains with moderate relief,” together accounting for more than 88% of Highly Suitable areas. These regions, characterized by complex terrain and varied topography, promote favorable microclimates for UMEBLF. Portions of Highly and Moderately Suitable habitat also occur in “mid‐elevation mountains with moderate relief” and “mid‐ to high‐elevation mountains with small relief,” further emphasizing that UMEBLF sensitivity to terrain is not reflected simply in absolute elevation but is closely related to the geomorphological settings along the pathways of warm and humid airflows.

## DISCUSSION

### Advantages of machine learning for suitable habitat simulation and prediction

Accurately characterizing the suitable distribution of UMEBLF under small‐sample conditions constitutes a typical nonlinear ecological modeling challenge (Elith & Leathwick, 2009; Franklin, 2010). In this study, machine learning algorithms were applied to a forest type shaped by complex local interactions between climate and terrain, thereby overcoming the limitations of traditional statistical models in representing nonlinear relationships among multiple environmental drivers (Austin, 2002). Compared with conventional statistical approaches and widely used tools such as MaxEnt and Biomod (Phillips et al., 2006; Thuiller et al., 2019), the RF model is more effective at capturing nonlinear relationships and interaction effects among environmental predictor variables. This characteristic makes RF particularly suitable for forest types strongly controlled by local climate–topography coupling mechanisms (Breiman, 2001; Cutler et al., 2007; Olden et al., 2008). The 54 presence samples used in this study adequately represented the major environmental gradients associated with UMEBLF suitable habitats, and the RF model showed good robustness. These results are consistent with recent studies on forest suitability modeling in regions with complex terrain (Lee et al., 2022; Wu et al., 2025).

Model predictions show strong agreement with validation samples and align well with existing ecological understanding of UMEBLF spatial distribution patterns. Validation using independent third‐party ecological data, namely the montane cloud–fog belt, further confirms that RF‐based mapping of suitable UMEBLF habitats closely reflects real‐world conditions (Beery et al., 2021). Together, these results demonstrate the strong applicability of machine learning approaches for simulating UMEBLF habitat suitability. The framework presented here provides a useful reference for vegetation mapping and conservation planning based on artificial intelligence and geospatial big data while substantially reducing the time and labor demands of traditional field surveys assisted by remote sensing.

### Interpretation based on the cloud–fog belt

Visual comparison between the cloud–fog belt and the Optimally Suitable areas provides a climate‐based explanation for the reliability of RF model predictions (Foster, 2001; Hamilton et al., 1995; Schratz et al., 2018). UMEBLF distribution exhibits a strong spatial correspondence with the cloud–fog belt, with Optimally Suitable areas mainly located on the middle and upper windward‐side slopes of mountains under the influence of the southwest monsoon.

During the rainy season, warm and humid air masses advance upslope, forming temperature inversion layers and frequent fog condensation. These processes collectively form a stable cloud–fog belt that acts as a persistent thermal buffer, providing the microclimatic conditions necessary for UMEBLF persistence (Bruijnzeel, Scatena, & Hamilton, 2011; Still et al., 1999). This evidence indicates that UMEBLF distribution is not primarily an adaptation to low temperatures. Rather, the consistently warm and humid conditions generated by the cloud–fog belt support the growth of UMEBLF species (Bubb et al., 2004). Moreover, because the overlapping belts identified here do not include inversion effects caused by valley winds, the results further highlight the fact that monsoon‐driven inversion is a key mechanism enabling the existence of UMEBLF (Muñoz‐Villers et al., 2012).

Optimally Suitable areas are most concentrated along the Cangshan–Ailao Mountain line, with most areas located to the west of this boundary. Warm and humid air masses originating from the Indian Ocean move northward into central Yunnan and interact strongly with windward mid‐montane slopes. The northwest–southeast orientation and high elevations of the Ailao Mountains, with peaks reaching 3166 m, effectively obstruct eastward movement of the southwest monsoon. After crossing the ridge, air masses descend and accumulate on leeward slopes and enter the northeastern mid‐montane region of the Ailao Mountains. The resulting distribution appears as fragmented bands rather than continuous forest distributions (Bruijnzeel, Scatena, & Hamilton, 2011). Moreover, the oblique orientation of the Ailao Mountains relative to the direction of the southwest monsoon causes a slight northward deflection of airflow, resulting in Optimally Suitable areas that broadly follow contour lines while exhibiting a slight northward shift.

### Climatic and topographic drivers of UMEBLF distribution

Variable importance analysis combined with distribution statistics indicates that temperature seasonality (BIO4), DEM, and DMTZ are the primary factors shaping UMEBLF spatial distribution (Austin, 2002; Ge et al., 2019; Koç et al., 2018; Koç et al., 2024). Repeated subsample modeling further indicated that temperature seasonality (BIO4), DEM, DMTZ, and MRAI consistently made relatively high contributions under different presence‐sample subsampling levels. Among these, temperature seasonality (BIO4) has the highest importance in the RF model, highlighting the decisive role of intra‐annual temperature variability in determining habitat suitability (Álvarez‐Álvarez et al., 2025). Lower temperature seasonality reflects more stable thermal conditions throughout the year, which favor evergreen broad‐leaved species by supporting continuous growth cycles and are consistent with the ecological requirements of UMEBLF.

Elevation, represented by DEM, does not act through height alone but serves as an indicator of combined thermal and moisture gradients. Optimally Suitable areas are concentrated in the mid‐montane zone rather than in low‐elevation basins or high, cold mountains, indicating clear upper and lower temperature thresholds for UMEBLF distribution (Dobrowski, 2011a). DMTZ further influences the spatial distribution of suitable habitats by influencing airflow pathways, cloud formation, and microclimate maintenance, with Optimally Suitable areas primarily associated with mid‐ to high‐elevation mountains characterized by large or moderate relief (Gallant & Dowling, 2003; Jasiewicz & Stepinski, 2013; Suggitt et al., 2011).

Overall, UMEBLF distribution is not controlled by a single climatic variable or absolute elevation but results from the combined effects of thermal stability, relative topographic position, and geomorphological structure. Although specific thresholds and spatial extents vary with mountain orientation and windward or leeward conditions, this composite mechanism is consistent across mountain systems (Dobrowski, 2011a; Körner, 2007). This stability‐oriented climatic constraint distinguishes UMEBLF from other subtropical forest types. Rather than favoring high temperatures and high rainfall, UMEBLF thrives under conditions characterized by constrained minimum temperatures, low annual temperature variability, sufficient dry‐season precipitation, and limited moisture seasonality.

### Implications of future warming for UMEBLF

UMEBLF shows relatively strong adaptability to warming climates. Under low‐emission scenarios, the distribution of suitable habitats remains relatively stable. Under medium‐ and high‐emission scenarios, however, Optimally Suitable and Highly Suitable areas gradually decline, particularly along habitat margins. This pattern of stable core areas and dynamic edges suggests a limited buffering capacity against warming while revealing increasing degradation risks under continued temperature rise and alterations in dry‐season precipitation.

Under the SSP5‐8.5 scenario, the Optimally Suitable area is projected to decrease to approximately 3.0 × 10^4^ km^2^ by the 2090s, accounting for only 7.7% of the region. Such degradation may weaken the carbon sequestration and water conservation functions of the forest and exacerbate habitat fragmentation for wildlife in marginal areas, warranting particular attention in future ecological management and biodiversity conservation strategies (Mahmoud et al., 2025).

### Conservation implications

Current nature reserves encompass most Optimally Suitable UMEBLF areas, particularly within the core zones of the Ailao, Wuliang, and Gaoligong Mountains and the Lincang Lancangjiang region (Qian et al., 2020). Nevertheless, some Optimally Suitable habitats remain outside reserve boundaries, and future climate scenarios indicate the emergence of new suitable areas along habitat margins. These regions represent potential conservation gaps.

Consistent with recommendations from previous studies on conservation gaps (Brennan et al., 2022; Ji et al., 2022; Li et al., 2024; Mi et al., 2023; Xu et al., 2024), future UMEBLF conservation should prioritize forest protection, monitoring, and restoration within Optimally Suitable habitats located in reserve core zones, particularly in mid‐montane regions such as the Ailao and Gaoligong Mountains and the Lancangjiang area (Pressey et al., 2015). In addition, ecological buffer zones and habitat corridors should be established in marginal areas where suitability is projected to decline, thereby enhancing the resilience of UMEBLF communities to future climate change.

### Limitations and uncertainty

The UMEBLF suitability maps generated in this study represent theoretical distributions primarily driven by natural environmental factors (Lewis et al., 2015). In practice, many Optimally Suitable areas outside existing reserves have undergone forest degradation or land‐use change. For example, parts of the Ailao Mountains have been transformed into orchards, tea plantations, or construction land (Laurance et al., 2014; Xu et al., 2014), meaning that predicted suitability does not necessarily correspond to current forest presence (Shen et al., 2024; Xiong et al., 2022). In western Yunnan, although the Cangshan and Nujiang regions contain Optimally Suitable areas of 2733 and 1643 km^2^, respectively, observed UMEBLF is restricted to scattered locations such as Biluo Snow Mountain, with many original stands replaced by artificial forests (e.g., Yunnan pine and Huashan pine). Because large portions of original UMEBLF are located within nature reserves, suitable areas outside reserves are often occupied by secondary forests. Therefore, this study relied only on the 54 verified occurrence points and the cloud–fog belt for validation. Validation results showed that these presence samples had good representativeness of the environmental conditions across the whole study area and that RF models developed from different subsets also exhibited good robustness. However, owing to the banded, insular, and fragmented distribution pattern of UMEBLF itself, the limited sample size may still affect the identification of local edge habitats and small residual patches. Constrained by field accessibility, the lack of historical vegetation records, and limitations associated with forest regeneration and succession, future studies should combine higher resolution forest inventory data and remote sensing imagery to conduct downscaled spatial mapping, thereby reducing omission errors in marginal habitats.

The RF model, combined with mechanistic interpretation, has potential for cross‐regional generalization. Although this study focuses on Yunnan, similar climatic and topographic conditions occur in northern Laos, northeastern Myanmar, northern Vietnam, and northern Thailand, suggesting that the model may be applicable in these regions. Nonetheless, further refinement of predictor variables and systematic validation are required. Differences in vegetation classification and naming systems among countries may constrain direct cross‐border comparisons.

Additional uncertainties arise from variable selection and future climate scenario assumptions. Important ecological process variables, such as vegetation physiological responses and soil moisture dynamics, were not included (Mod et al., 2016). Future climate projections relied on a single general circulation model, which does not capture structural uncertainties among models (Knutti et al., 2010; Tebaldi & Knutti, 2007). The 1‐km spatial resolution may also underestimate microtopographic effects in complex mid‐montane terrain (Dobrowski, 2011b; Lembrechts et al., 2019). Finally, suitable area statistics were derived from vectorization of raster outputs, and the removal of small polygons and smoothing parameters may influence the results (Arnone et al., 2016).

## CONCLUSIONS

This study constructed and selected an optimal RF model to map the suitable distribution of UMEBLF using 11 environmental variables. The analysis identified the key drivers of UMEBLF habitat suitability and conducted integrated mapping and conservation assessments under both current and future climate scenarios. By moving beyond traditional field survey approaches supplemented by remote sensing imagery, this study provides a machine learning‐based framework for vegetation mapping and conservation gap analysis driven by geospatial big data.

The RF model exhibited the best performance in terms of predictive accuracy, stability, and ecological interpretability. UMEBLF suitability is jointly controlled by temperature seasonality, elevation, and geomorphological structure. Under future warming scenarios, marginal UMEBLF habitats face a markedly increased risk of suitability degradation, while Optimally Suitable areas that are currently well covered by nature reserves are projected to decrease in extent. Areas most sensitive to climate warming are primarily located in experimental zones along the peripheries of existing reserves.

Overall, this study provides both theoretical support and methodological reference for UMEBLF conservation and management. Future conservation planning should incorporate adaptive strategies to address climate change, including dynamic adjustment and the establishment of ecological corridors in marginal areas where degradation risks are pronounced. Further research should build on the framework proposed herein by incorporating additional anthropogenic drivers, expanding the range of climate scenarios, extending the spatial scope, and improving spatial resolution to enhance the accuracy of suitability mapping.

## AUTHOR CONTRIBUTIONS

Wenqi Zhang undertook the major tasks of this study, including research conception, methodology design, data processing, model construction, result analysis, and manuscript drafting. Yanxia Wang and Ruliang Zhou were responsible for overall guidance and supervision of the research, as well as for revising and finalizing the manuscript. Yuhang Zhou, Yuanbei Liu, and Weihong Zeng provided support in field investigations, data verification, and visualization. All authors contributed to the revision of the manuscript and approved the final version for publication.

## FUNDING INFORMATION

This research was supported by the National Natural Science Foundation of China (Grant No. 42061004), the Special Project for Building a Science and Technology Innovation Center for South and Southeast Asia, China (Grant No. 202503AP140004), and the Youth Special Project of Xing Dian Talent Support Program of Yunnan Province, China (Grant No. XDYC‐QNRC‐2022‐0230).

## CONFLICT OF INTEREST STATEMENT

The authors declare no conflicts of interest.

## Supporting information


Appendix S1.

## Data Availability

Data and code (Zhang, 2026) are available in Zenodo at https://doi.org/10.5281/zenodo.21943995.
