# Supplementary material for: Mapping and conservation analysis of upper montane evergreen broad‐leaved forest using machine learning and geodata
Source: Ecol Appl. 2026 Oct 22;36(7):e70333. doi: 10.1002/eap.70333 (PMC13691411; doi:10.1002/eap.70333)
Supplement: Supplementary file 1 [file EAP-36-e70333-s001.pdf]

Appendix S1

Mapping and conservation analysis of upper montane evergreen broad-leaved forest using machine learning and geodata

Wenqi Zhang, Yanxia Wang, Yuhang Zhou, Yuanbei Liu, Weihong Zeng, Ruliang Zhou

Ecological Applications

Table S1 Area and proportion of different geomorphological types under each suitability level.

Geomorphological Type	Optimally Suitable		Highly Suitable		Moderately Suitable		Marginally Suitable		Total	
	Area(km^2)	Proportion within class(%)	Area(km^2)	Proportion within class(%)	Area(km^2)	Proportion within class(%)	Area(km^2)	Proportion within class(%)	Area(km^2)	Proportion within province(%)
Low-elevation hills	0	0.00%	221	0.19%	2,135	1.30%	84	0.08%	2,440	0.56%
Low-elevation low-relief mountains	0	0.00%	265	0.22%	1,135	0.69%	3,100	2.95%	4,500	1.04%
Low-elevation moderate-relief mountains	0	0.00%	2	0.00%	37	0.02%	75	0.07%	114	0.03%
Mid-elevation hills	10	0.02%	321	0.27%	1,171	0.71%	833	0.79%	2,335	0.54%
Mid-elevation low-relief mountains	86	0.19%	1,304	1.10%	8,480	5.17%	20,065	19.12%	29,935	6.90%
Mid-elevation moderate-relief mountains	1,224	2.64%	10,396	8.77%	32,569	19.87%	31,201	29.72%	75,390	17.38%
Mid-elevation high-relief mountains	114	0.25%	2,458	2.07%	5,782	3.53%	4,881	4.65%	13,235	3.05%
Mid-to-high elevation hills	54	0.12%	982	0.83%	1,599	0.98%	2,739	2.61%	5,374	1.24%
Mid-to-high elevation low-relief mountains	484	1.05%	4,675	3.94%	9,962	6.08%	8,307	7.91%	23,428	5.40%
Mid-to-high elevation moderate-relief mountains	3,689	7.97%	13,699	11.55%	26,194	15.98%	18,891	18.00%	62,473	14.40%
Mid-to-high elevation high-relief mountains	37,064	80.08%	70,044	59.07%	44,429	27.11%	7,934	7.56%	159,471	36.77%
Mid-to-high elevation very high-relief mountains	1,546	3.34%	1,672	1.41%	2,048	1.25%	204	0.19%	5,470	1.26%

High-elevation high-relief mountains	34	0.07%	1,005	0.85%	8,053	4.91%	2,178	2.07%	11,270	2.60%
High-elevation very high-relief mountains	630	1.36%	5,300	4.47%	7,302	4.46%	487	0.46%	13,719	3.16%
Very high-elevation very high-relief mountains	0	0.00%	2	0.00%	255	0.16%	1	0.00%	258	0.06%
Mid-to-high elevation loess ridges and mounds	0	0.00%	30	0.03%	61	0.04%	0	0.00%	91	0.02%
Low-elevation alluvial–proluvial terrace	0	0.00%	2	0.00%	33	0.02%	159	0.15%	194	0.04%
Mid-elevation alluvial–proluvial–lacustrine terrace	0	0.00%	14	0.01%	71	0.04%	151	0.14%	236	0.05%
Mid-elevation alluvial–proluvial terrace	63	0.14%	293	0.25%	455	0.28%	0	0.00%	811	0.19%
Mid-elevation lava accumulation terrace	292	0.63%	251	0.21%	391	0.24%	0	0.00%	934	0.22%
High-elevation erosional terrace	73	0.16%	24	0.02%	56	0.03%	3	0.00%	156	0.04%
Low-elevation alluvial–proluvial plain	0	0.00%	1,127	0.95%	2,770	1.69%	293	0.28%	4,190	0.97%
Mid-elevation proluvial–lacustrine plain	149	0.32%	1,136	0.96%	273	0.17%	259	0.25%	1,817	0.42%
Mid-elevation alluvial plain	8	0.02%	235	0.20%	178	0.11%	9	0.01%	430	0.10%
Mid-elevation alluvial–proluvial plain	553	1.19%	1,274	1.07%	3,207	1.96%	2,319	2.21%	7,353	1.70%

Mid-to-high elevation proluvial–lacustrine plain	118	0.25%	470	0.40%	1,127	0.69%	156	0.15%	1,871	0.43%
Mid-to-high elevation alluvial plain	0	0.00%	77	0.06%	149	0.09%	1	0.00%	227	0.05%
Mid-to-high elevation alluvial–proluvial plain	83	0.18%	775	0.65%	3,103	1.89%	549	0.52%	4,510	1.04%
Modern glacier	1	0.00%	70	0.06%	343	0.21%	35	0.03%	449	0.10%
Lake	11	0.02%	454	0.38%	522	0.32%	53	0.05%	1,040	0.24%

This table summarizes the area of each suitability level (Optimally Suitable, Highly Suitable, Moderately Suitable, and Marginally Suitable) within different geomorphological types (DMTZ), as well as their proportions within each geomorphological category.

The table supplements the analysis in the main text regarding the influence of geomorphological type as a categorical variable on the suitable distribution of UMEBLF, providing quantitative support for the differentiation of suitability patterns under different geomorphological backgrounds.
